# The Role of Micronutrient Deficiencies in Nutritional Anemia Beyond Iron: An Expert Perspective on the Scope and Strength of the Current Evidence^☆^

**DOI:** 10.1016/j.advnut.2026.100697

**Published:** 2026-07-03

**Authors:** Colleen C Farrell, Jason L Burkhead, James F Collins, Alison D Gernand, Tim J Green, Leane Hoey, Mihaela C Kissell, Helene McNulty, Alvaro F Perez, Manuel Ruz, Maret G Traber, Crystal D Karakochuk

**Affiliations:** 1 Food, Nutrition and Health, Faculty of Land and Food Systems, The University of British Columbia, Vancouver, BC, Canada; 2 BC Children’s Hospital Research Institute, and Women’s Health Research Institute, Vancouver, BC, Canada; 3 Institute of Arctic Biology, University of Alaska Fairbanks, Fairbanks, AK, United States; 4 Food Science & Human Nutrition Department, University of Florida, Gainesville, FL, United States; 5 Department of Nutritional Sciences, Penn State University, University Park, PA, United States; 6 College of Nursing and Health Sciences, Flinders University, Adelaide, SA, Australia; 7 Nutrition Innovation Centre for Food and Health (NICHE), School of Biomedical Sciences, Ulster University, Coleraine, Northern Ireland; 8 Healthy Mothers Healthy Babies Consortium, Micronutrient Forum, Washington, DC, United States; 9 Department of Nutrition, Faculty of Medicine, University of Chile, Santiago, Chile; 10 Linus Pauling Institute, Oregon State University, Corvallis, OR, United States

**Keywords:** anemia, hemoglobin, iron, micronutrient, mineral, review, vitamin

## Abstract

There is a tacit assumption that the majority of nutritional anemia is caused by iron deficiency. However, several other key micronutrients are involved in iron and/or erythrocyte metabolism and thus play a role in the development of nutritional anemia. This consensus paper provides an updated review that synthesizes current evidence on the role of individual micronutrients, beyond iron, in the development of nutritional anemia and identifies emerging hypotheses and potential mechanistic links to erythropoiesis and iron metabolism involving nutrients of both established and growing interest. To achieve this, we convened leading experts in micronutrients and nutritional anemia to categorize 10 key micronutrients of interest into a hierarchy based on the scope and strength of evidence for their roles in the development of anemia, erythropoiesis, and iron metabolism. Folate, vitamin B-12, and vitamin A were ranked as having strong evidence, meaning data are consistent in describing a causal association between nutrient status and anemia development, and the evidence for the proposed mechanism(s) is well established. Riboflavin, zinc, vitamin B-6, vitamin C, and vitamin E were ranked as having moderate evidence, and vitamin D and copper were ranked as having potential and/or emerging evidence. We also identify critical gaps in the current evidence and propose priority areas for future research for each micronutrient. This perspective paper aims to serve as a comprehensive reference to inform future research priorities and guide global nutrition policy, strategy, and programming related to anemia prevention and control.

**Statement of significance**

This expert perspective paper provides an updated review of 10 key micronutrients contributing to nutritional anemia (beyond iron), including those with emerging mechanistic links. By categorizing each of the micronutrients according to the scope and strength of the evidence and highlighting the underrecognized biological pathways and their role in anemia development, this work provides an up-to-date comprehensive framework to guide research, policy, and holistic interventions for anemia prevention and control globally.

## Introduction

Anemia is a condition in which the number of red blood cells (RBCs) or the hemoglobin concentration, the oxygen-carrying compound within RBCs, is lower than normal and is insufficient to meet the body’s physiologic needs [1]. Anemia is typically identified when hemoglobin concentration is below a threshold determined by an individual’s age, sex, pregnancy status, and altitude of residence (above 1000 m) [1]. The etiology of anemia is multifactorial, encompassing infection and inflammation [2], disease [3], and genetic hemoglobin disorders [4]. Nutritional anemia is one of the most common forms, where the anemia is a consequence of nutritional deficiencies, inadequate diet, or insufficient absorption of nutrients [5]. Iron deficiency is the leading contributor to nutritional anemia [6], and nutrition-specific interventions using iron supplementation have been shown to increase hemoglobin concentrations and reduce risk of iron deficiency anemia (IDA) in certain populations [7]. However, deficiencies of several other key nutrients involved in iron and/or erythrocyte metabolism, beyond iron, may also contribute to the development of nutritional anemia [1].

**FIGURE 1:**
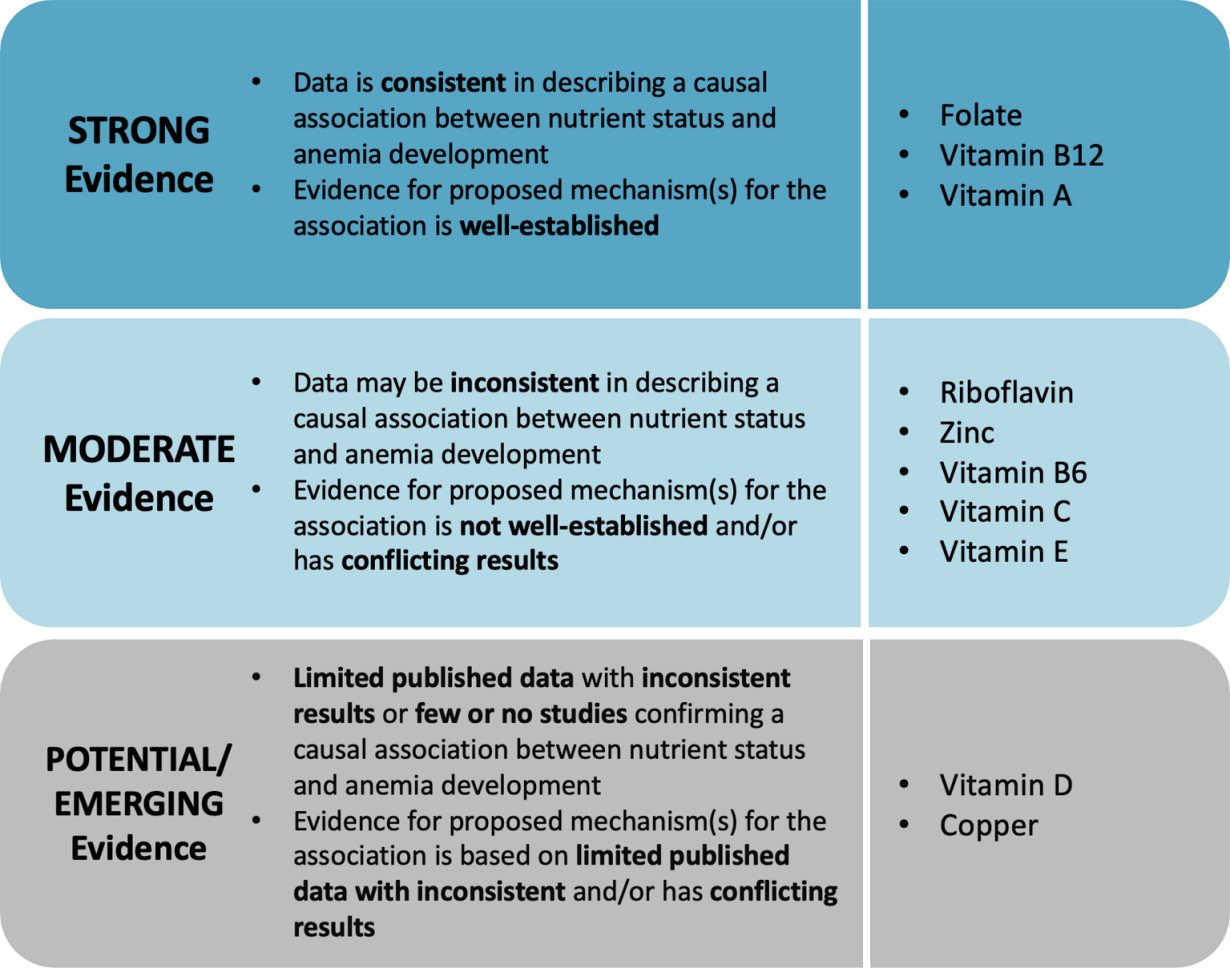
Classification of the current state of evidence of nutrients involved in nutritional anemia. “Strong evidence” is defined as published studies (e.g., randomized controlled trials, systematic reviews, and meta-analyses) that are consistent in describing a causal association between the nutrient status of interest and the development of anemia, and the evidence for the proposed mechanism(s) for the association is well established in human studies (e.g., broad consensus in the field and repeated studies showing consistent results). “Moderate evidence” is defined as published studies that may be inconsistent or variable in establishing a causal association between the nutrient status and development of anemia, and the evidence for proposed mechanisms for the association is not well established and/or has conflicting results in human studies (e.g., there are gaps in the current evidence to establish a causal role). “Potential/emerging evidence” is defined as there being limited published studies with inconsistent or variable results or there being few/no studies that confirm a causal association between nutrient status and anemia development, and the evidence for proposed mechanism(s) for the association is based on limited published studies with inconsistent, speculative, and/or conflicting results.

Over 2 decades ago, Fishman et al. [8] published a landmark paper that described the role of vitamins in the prevention and management of nutritional anemia. Since this publication, there has been a plethora of research that has identified new biological mechanisms and causal associations, further strengthening the state of the evidence for certain nutrients and underrecognized nutrients (e.g., vitamin D, zinc, and copper), which have been proposed as playing potentially critical roles in the development of anemia. The objective of this expert perspective paper was to provide an updated review of the existing literature on the role of various micronutrients, beyond iron, known to contribute to the development of nutritional anemia and to provide an expert perspective on the scope and strength of the current evidence (categorized as strong, moderate, or potential and/or emerging). The key vitamins described in the landmark Fishman et al. [8] review (i.e., riboflavin, folate, vitamin A, vitamin B-6, vitamin B-12, vitamin C, and vitamin E) were included in this review, as well as 3 other nutrients (i.e., zinc, vitamin D, and copper) that have gained growing recognition for their potential role in the development of nutritional anemia (Figure 1).

We also aimed to summarize the proposed mechanisms of nutrient deficiencies in the role of nutritional anemia (Table 1) and highlight any new or emerging hypotheses and/or biological mechanisms for the micronutrients of interest, beyond iron.

**TABLE 1:** Proposed mechanisms of nutrient deficiencies underlying the development of anemia

Nutrient	Potential mechanisms	Contribution to anemia
Folate	⁃ Impaired DNA synthesis ⁃ Impaired cell division	Impaired erythropoiesis resulting in large, immature RBC precursors (megaloblasts) in the bone marrow and macrocytes in the blood
Vitamin B-12	⁃ Impaired folate metabolism ⁃ Impaired DNA synthesis	
Vitamin A	⁃ Impaired iron mobilization affecting the storage, transport, and use of iron	Iron-restricted erythropoiesis
	⁃ Impaired immune function	Increased susceptibility to disease and/or infection; increasing potential risk of “anemia of inflammation”
Riboflavin	⁃ Impaired iron absorption or mobilization	Impaired erythropoiesis and release of ferritin-bound iron
	⁃ Increased gastrointestinal iron losses	Iron-restricted erythropoiesis
	⁃ Impaired vitamin B-6 synthesis as FMN is required for the generation of PLP in tissues (the active form of vitamin B-6)	Indirect contribution to anemia via vitamin B-6 deficiency
Zinc	⁃ Impaired zinc-dependent enzyme activity in the heme biosynthesis pathway ⁃ Decreased activity of zinc finger transcription factors ⁃ Reduced IGF-1 levels	Reduced erythropoiesis
	⁃ Reduced antioxidant activity	Increased RBC hemolysis
Vitamin B-6	⁃ Impaired enzyme activity in heme biosynthesis	Heme-restricted erythropoiesis
Vitamin C	⁃ Impaired intestinal iron absorption and iron mobilization from body stores ⁃ Impaired folate metabolism ⁃ Altered expression of erythrocyte receptors	Reduced erythropoiesis
	⁃ Oxidative damage to erythrocytes	RBC hemolysis
	⁃ Reduced collagen production	Blood loss and secondary IDA
Vitamin E	⁃ Oxidative stress and cell damage	Damaged erythrocytes and hemolysis
Vitamin D	⁃ Impaired maturation of erythroid precursor cells in the bone marrow	Ineffective erythropoiesis
	⁃ Impaired immune function	Increased susceptibility to disease and/or infection; increasing potential risk of “anemia of inflammation”
Copper	⁃ Decreased absorption of dietary and supplemental iron	Hypoferremia leading to iron-restricted erythropoiesis and anemia
	⁃ Impaired release of storage iron	
	⁃ Impaired mitochondrial function in hematopoietic stem cells	Reduced number of mature RBCs
	⁃ Reduced heme biosynthesis in erythroid precursors	Low erythrocyte heme content

Abbreviations: FMN, flavin mononucleotide; IDA, iron deficiency anemia; IGF-1, insulin-like growth factor-1; PLP, pyridoxal 5’-phosphate; RBC, red blood cell.

## Methods

### Expert group formation

The conceptual framework and scope of the paper were designed by 4 of the authors (CDK, CCF, HM, and ADG), who subsequently identified and invited contributing authors on the basis of their recognized and established work in the 10 micronutrients of interest.

### Nutrients of interest selection

This review covers the same vitamins described in the Fishman et al. [8] publication—riboflavin, folate, vitamin A, vitamin B-6, vitamin B-12, vitamin C, and vitamin E—and extends this scope to include 3 additional micronutrients: zinc, vitamin D, and copper. These additional nutrients were agreed upon by a consensus among all authors, reflecting the growing body of evidence on the potential roles of these micronutrients in the development of nutritional anemia since the Fishman et al. [8] publication.

### Population and scope

No restriction was placed on population group or life stage.

### Review process and evidence classification

A concept note was developed and agreed upon by all authors, establishing a clear framework to evaluate and classify the current state of evidence. Each author was responsible for summarizing the best available evidence for their micronutrient of specialization and prioritizing the highest quality evidence [e.g., systematic reviews and meta-analyses and randomized controlled trials (RCTs)]. Although there was no formal restriction placed on publication date, data published after the Fishman et al. [8] article were prioritized to be included in this review.

To ensure consistency, each nutrient was classified into a hierarchy of strength and quality of evidence on the basis of the following criteria, applied and agreed upon by all authors:

⁃ Strong evidence: strong and/or high-quality evidence where the published data are consistent in describing a causal association between nutrient status and the development of anemia (e.g., RCTs) and/or the evidence for the proposed mechanism(s) for the association is well established.
⁃ Moderate evidence: medium and/or moderate-quality evidence where the published data may be inconsistent in showing a causal association between nutrient status and the development of anemia and/or the evidence for the proposed mechanism(s) for the association is not well established and/or has conflicting results.
⁃ Potential or emerging evidence: weak and/or low-quality evidence where there is limited published data with inconsistent results, few or no studies confirming a causal association between nutrient status and the development of anemia, and/or the proposed mechanism(s) for the association is based on limited published data with inconsistent and/or conflicting results.

Sections were reviewed by lead authors (CCF and CDK) to ensure consistency in the application of these criteria.

### Strong evidence (folate, vitamin B-12, and vitamin A)

#### Folate

##### Functional effects

Folate is essential for one-carbon metabolism through transferring and using one-carbon units in key biological processes [9]. Folate exists in many cofactor forms, including 5,10-methylenetetrahydrofolate, which is required for pyrimidine biosynthesis, or it can be converted to other cofactor forms for purine biosynthesis, which are precursors of DNA. The cofactor form 5-methyltetrahydrofolate (5-MTHF) is required for the vitamin B-12–dependent conversion of homocysteine to methionine, which generates S-adenosylmethionine (SAM). SAM acts as a methyl donor for numerous methylation reactions, including the methylation of DNA and proteins [10]. Clinical folate deficiency manifests as megaloblastic anemia, characterized by megaloblasts in the bone marrow, hypersegmented neutrophils, and macrocytes in the peripheral blood [11].

##### Prevalence of deficiency

Comparing prevalence rates of deficiency between studies or countries is challenging because the assays used to assess folate status and the cutoff values used to evaluate deficiency are inconsistent. Further, exposure to folic acid (the synthetic form of the vitamin used in dietary supplements and food fortification) differs on the basis of mandatory and/or voluntary fortification practices of the local food supply [10]. In countries with mandatory fortification, the prevalence of folate deficiency is almost nonexistent (Supplementary Table 1) [[12], [13], [14]]. In a systematic review of females of reproductive age (aged 12‒49 y), the prevalence of deficiency was typically found to be >20% in lower-income countries and <5% in higher-income countries [15]. Low folate status has also been reported among adults [16,17], preschool children [18], and pregnant females, but evidence is greatly lacking [10].

##### Key evidence for the role of folate in nutritional anemia

Several trials in various populations have investigated whether the addition of folic acid (or other folate forms), compared with iron alone, can further improve hematologic responses or reduce anemia [[19], [20], [21], [22], [23], [24]] (Supplementary Table 2).

In Brazilian children aged 6 to 24 mo, combined supplementation (iron plus folic acid) was more effective at lowering anemia prevalence than iron only (35% compared with 14% reduction after 3 mo of intervention), although both interventions were equally effective in improving mean hemoglobin concentrations [22]. Conversely, a study in Mexican children of a similar age reported that combined administration of iron with folic acid was no more effective than iron alone in improving hemoglobin concentrations and reducing the prevalence of anemia [23]. In a trial from India, in which hemoglobin was measured as a secondary outcome, folic acid supplementation for 6 mo had no effect on hemoglobin concentrations in children aged 6 to 30 mo [24]. However, nonsevere anemia was present in 70% of the children at baseline, and despite treatment with iron for the initial 2 mo, nearly 90% of these children remained anemic at the end of the intervention period. This suggests that other nutrient deficiencies, or non-nutritional factors, may be causing or contributing to anemia.

Among pregnant females from Mexico with IDA and normal serum folate concentrations at baseline, significantly greater improvements in hemoglobin concentrations were observed in females given the combined treatment containing folinic acid and iron than in females given iron alone [20]. In a study of pregnant females in rural Nepal, combined supplementation of folic acid and vitamin A from 10 weeks of gestation did not slow down the expected decline in hemoglobin concentrations or reduce risk of anemia at 32 weeks of gestation or 6 wk postpartum compared with vitamin A alone [21]. Iron status may have limited the hematologic response in the folic acid group because IDA was present in the third trimester in 52% of the females given folic acid, compared to 12% to 18% in the additional intervention groups who were also exposed to iron. These findings generally support those from a Cochrane review of folic acid supplementation studies in pregnancy, which concluded that supplementation had no benefit on hematologic markers [25].

In early postpartum females with normal folate status, no significant differences in hematologic parameters or anemia prevalence were observed in those who received supplementation with iron and ascorbic acid compared with iron, ascorbic acid, and folic acid; females in both intervention groups had significantly higher hemoglobin, hematocrit, and erythrocyte counts compared with the unsupplemented group [19].

Historically, there was some concern that chronic exposure to high-dose folic acid could “mask” the anemia of vitamin B-12 deficiency [26]. More recently, other health concerns have been raised, including evidence to suggest that low vitamin B-12 status combined with high folate concentrations may be associated with anemia [27] or could exacerbate metabolic markers of vitamin B-12 deficiency [28,29], compared with those with low vitamin B-12 combined with normal folate status. The limited evidence is, however, observational and generally inconclusive [30].

##### Gaps in current evidence and future research areas

The current evidence for folate has been summarized from studies conducted in a limited number of population subgroups with mild anemia, if present, and generally with normal folate status at baseline. The aforementioned studies used serum/plasma folate (reflects recent dietary intake rather than tissue stores) and different methodologies to measure folate status [19,[22], [23], [24]], were limited by small sample size [19,[22], [23], [24]] and duration of <3 mo [19,20], or were not designed with hematological parameters as a primary outcome [24]. Further studies are required in population groups at risk of anemia who also have suboptimal folate status and lower hemoglobin concentrations at baseline.

#### Vitamin B-12

##### Functional effects

Vitamin B-12 (cobalamin) is required as a cofactor for 2 enzymes, 1 of which is closely interrelated to folate in DNA synthesis and hematopoiesis [31]. In one-carbon metabolism, the enzyme methionine synthase requires vitamin B-12 (in the form of methylcobalamin) for the remethylation of homocysteine to methionine, the precursor of SAM. In this reaction, 5-MTHF is also demethylated to tetrahydrofolate. If vitamin B-12 is deficient, methionine synthase activity is decreased, leading to elevated homocysteine and “trapping” of folate in the form of 5-MTHF. As a result, the cofactor form 5,10-methylenetetrahydrofolate is depleted, leading to impaired synthesis of thymidine. A reduced supply of thymidine can affect DNA synthesis, potentially leading to megaloblastic anemia [31].

##### Prevalence of deficiency

Measurement and comparison of vitamin B-12 deficiency prevalence rates across studies are challenging because different biomarkers or combinations of biomarkers are used, and for any 1 biomarker, no universally accepted cutoff value is used to define deficient status [31]. Nevertheless, vitamin B-12 deficiency is common worldwide, and this varies by country, population studied, and method of measurement (Supplementary Table 3). Pregnant individuals [12,32] and older adults [13,33] are considered to be at greatest risk of vitamin B-12 deficiency. Higher prevalence is also reported in vegetarians [34], with vegans found to be at highest risk of vitamin B-12 deficiency [35], which is not surprising considering that there are typically no plant sources of vitamin B-12 in human diets.

Severe vitamin B-12 deficiency, called pernicious anemia, is an autoimmune disorder caused by loss of intrinsic factor, the glycoprotein that facilitates the absorption of vitamin B-12 in the terminal ileum [31]. Although the incidence of pernicious anemia increases with age, it is relatively uncommon, estimated to affect ∼2% to 3% of adults aged >65 y [36,37].

##### Key evidence for the role of vitamin B-12 in nutritional anemia

Several studies have investigated whether the addition of vitamin B-12 improves the hematologic response obtained with iron and folic acid (IFA) supplementation [24,[38], [39], [40], [41], [42], [43], [44]] (Supplementary Table 4). In young children with anemia in India, supplementation with IFA and vitamin B-12 increased hemoglobin by 69% compared with 43% with IFA treatment alone [39]. Conversely, other studies from India in adolescent girls found that the addition of vitamin B-12 to IFA was less effective than IFA alone in improving hemoglobin concentrations and reducing the prevalence of anemia when administered daily [43] or had no further benefit when administered weekly [42]. The likely explanation for such inconsistent findings is that anemia prevalence at baseline is a key factor that differentiates the efficacy of vitamin B-12 supplementation, as the adolescent girls had mild-to-moderate anemia [42,43], whereas in the study by Chandelia et al. [39], the children had moderate-to-severe anemia and would more likely benefit from intervention. In the study by Gupta et al. [43], baseline vitamin B-12 status was significantly higher, and the prevalence of deficiency was lower in the vitamin B-12 plus IFA group compared with the IFA group (22% compared with 46% had vitamin B-12 concentrations <150 pmol/L, respectively). Additionally, rates of noncompliance and participant dropout were high. Therefore, the differences in micronutrient status and the prevalence of anemia at baseline may have limited the responses to vitamin B-12 intervention in these trials.

In studies where the intervention did not include IFA, vitamin B-12 had no effect on hematologic parameters in 6-wk-old infants with vitamin B-12 deficiency [41], in young children [24], in nonpregnant lacto-vegetarian females [38], in healthy females [40], or when vitamin B-12 was delivered in food-based interventions to both children and adults [44]. These studies were, however, limited by no control group [41], small sample size and short study duration [38], absence of anemia at baseline [40,41,44], or hematologic responses that were not a primary outcome [24,38,41,44]. A systematic review [45] and meta-analysis [46] in older adults concluded no beneficial effect of vitamin B-12 on anemia, as did a recent meta-analysis in pregnant females and their offspring [47]. Of note, these conclusions were based on a limited number of studies in each review paper, ranging from 2 to 4 trials.

In summary, vitamin B-12 studies were typically conducted among young children, adolescents, and females of reproductive age only and were mostly from India, a country with a high prevalence of vitamin B-12 deficiency [48]. Despite improvements in biomarkers of vitamin B-12 status with extremely high doses of the vitamin (125‒1000 *μ*g/d) compared with dietary levels, no benefit on hematologic indicators was observed, except in children aged <5 y with moderate-to-severe anemia.

##### Gaps in current evidence and future research areas

The current evidence is summarized from studies conducted in a limited number of population subgroups, mostly with mild-to-moderate or no anemia at baseline, which may have weakened any response to vitamin B-12 treatment. In the few studies that reported the prevalence of anemia, this remained high (∼60%) despite treatment with vitamin B-12 in addition to IFA [42,43], suggesting that other nutrients or non-nutrient factors may be an underlying cause of anemia. Differences in study design, including the use of various forms and doses of vitamin B-12, different routes, and frequency of administration, are factors that may have limited a potential hematologic response. Furthermore, although direct (serum total vitamin B-12 and holotranscobalamin) and functional (methylmalonic acid and homocysteine) biomarkers are available, only serum total vitamin B-12 is measured in most studies [49]. Given the limitations of each of the 4 vitamin B-12 biomarkers (e.g., thresholds for deficiency diagnosis, costs and availability, and factors confounding their accurate measurement), a combination of 2 or more biomarkers is ideally recommended to diagnose deficiency [50,51]. Given the limited and poor-quality evidence available, more well-designed intervention trials are required in at-risk population groups, especially in older adults who are at greatest risk of subclinical deficiency. Consensus must also be reached on how to define low and/or deficient vitamin B-12 status.

#### Vitamin A

##### Functional effects

Vitamin A is involved in a wide range of functions in humans, including cell growth and differentiation, vision, immune function, reproductive function, and iron metabolism and erythropoiesis [52]. The term vitamin A encompasses a class of retinoids, including retinyl esters and retinol (preformed vitamin A), β-carotene, and other carotenoids (provitamin A; converted to retinol during absorption and metabolism).

##### Prevalence of deficiency

Vitamin A deficiency is one of the most prevalent micronutrient deficiencies globally, disproportionately affecting young children and pregnant and lactating females, and is considered to be one of the top modifiable risk factors for the global burden of disease [53,54]. A third of children aged <5 y and 15% of pregnant females around the world are estimated to have a serum retinol concentration <0.70 *μ*mol/L (subclinical vitamin A deficiency), with the highest prevalence in countries in Africa and Asia [55]. In settings where vitamin A deficiency remains a public health concern, the WHO recommends vitamin A supplementation for children aged 6 to 59 mo [56]. A recent Cochrane review of 19 trials (∼1.2 million participants) found that vitamin A supplementation in children aged 6 mo to 5 y was associated with a 12% reduction in all-cause mortality and mortality due to diarrhea compared with the control group [56]. Although rates of deficiency have decreased over the past 20 y, many countries lack current national data on deficiency, limiting our ability to estimate the true burden worldwide [54,57].

##### Key evidence for the role of vitamin A in nutritional anemia

Numerous studies have found a positive correlation between retinol and hemoglobin [58]. Vitamin A deficiency is associated with lower mobilization of iron stores, resulting in more storage of iron in the liver and a similar iron biomarker profile as seen in iron deficiency, except for higher serum ferritin (storage), even if iron is not depleted [59]. In Morocco, a setting with a very high prevalence of vitamin A deficiency, children who received high-dose vitamin A (200,000 IU) compared with placebo had a decrease in both serum ferritin and transferrin receptor concentrations, whereas iron status (body iron stores) did not change [60]. Erythropoiesis is stimulated in part by a hormone called erythropoietin (EPO), which is produced in the kidneys. The *EPO* gene contains a retinoic acid response element for which retinoic acid is a ligand. Retinoic acid signaling has long been recognized for its importance in erythropoiesis, including in the fetal liver (the main site for EPO production during fetal development) [61]. Among Moroccan children, 2 large doses of vitamin A increased mean corpuscular volume and EPO concentrations [60].

Other potential mechanisms for the link between vitamin A and anemia include the influence of vitamin A on iron absorption and infection. Some have hypothesized that vitamin A can improve iron absorption by inhibiting the impact of phytates, etc., during digestion, although results are mixed in studies testing vitamin A and iron absorption directly [62,63]. Further, whereas infectious disease can impair erythropoiesis and iron utilization and vitamin A can improve immune function and reduce risk of infectious disease, few studies have directly examined the relationship between vitamin A deficiency and anemia of infection [58].

Trials of vitamin A supplementation have demonstrated the beneficial effect of vitamin A on reducing anemia, albeit not consistently. In a 2015 Cochrane review, maternal vitamin A supplementation compared with placebo or no treatment (3 trials; 15,649 participants) reduced risk of maternal anemia by 36% [95% confidence interval (CI): 6%, 57%] [64]. The evidence was rated as “moderate quality.” However, vitamin A with other micronutrients compared with micronutrients without vitamin A (3 trials; 706 participants) did not have the same benefit (risk ratio: 0.86; 95% CI: 0.68, 1.09), and there was no benefit of maternal vitamin A supplementation on neonatal anemia. In settings where vitamin A deficiency is common, vitamin A supplementation given with iron (and folic acid as the standard of care) increases hemoglobin concentrations and reduces maternal anemia [64]. Supplementation trials in infants and children are heterogeneous, and some examine the effects of vitamin A and iron together but not vitamin A alone. In a small but comprehensive study of vitamin A supplementation in school children in Morocco (*n* = 81), vitamin A improved hemoglobin and reduced anemia [60]. Over half of the children were anemic and over 3-quarters were vitamin A–deficient at baseline. A recent 2019 systematic review of vitamin A and iron status in children, pregnancy, and lactation found that supplementation reduced risk of anemia by 26% [65]. Improvements in iron status measured by serum ferritin were found in pregnancy and lactation but not in childhood and adolescence. In a review of neonatal vitamin A supplementation trials, only 1 reported hemoglobin as an outcome and did not find an effect [66]

Although few trials have tested vitamin A and iron in a factorial design and few measured biomarkers of both vitamin A and iron status at baseline and endline, those that do point to a bidirectional relationship (improvement of 1 with supplementation of the other) and the largest effect on reducing anemia when both are given together [58,59]. Notably, studies in pregnancy that saw improvements from supplementing with vitamin A compared with no vitamin A provided IFA to all participants as the standard of care. Overall, trials in children and females of reproductive age have shown that vitamin A supplementation, in populations with both vitamin A deficiency and anemia, typically increases hemoglobin by ∼2 to 12 g/L and sometimes by >20 g/L [58].

##### Gaps in current evidence and future research areas

Vitamin A supplementation and improved vitamin A status have the potential to reduce anemia through improving erythropoiesis and the use of existing body iron, particularly in populations with a high prevalence of both vitamin A deficiency and anemia; however, findings are inconsistent. Supplementation appears to be most effective when combined with iron. Future research should include clinical trials of vitamin A supplementation in which extensive vitamin A, iron, erythropoiesis, and inflammation biomarkers are concurrently measured over time.

### Moderate evidence (riboflavin, zinc, vitamin B-6, vitamin C, and vitamin E)

#### Riboflavin

##### Functional effects

The cofactor forms of riboflavin, flavin adenine dinucleotide (FAD), and flavin mononucleotide (FMN) are required in the metabolism of vitamin B-6, folate, vitamin B-12, and iron and in the synthesis of niacin from tryptophan. Thus, riboflavin deficiency causes perturbations in the functioning of these nutrients, potentially contributing to anemia [67].

The extent of riboflavin-related anemia can range in presentation from severe anemia with clinical deficiency to more moderate anemia with subclinical riboflavin deficiency [68]. Various mechanisms are proposed through which riboflavin deficiency could contribute to the development of anemia [69]. Riboflavin deficiency may impair iron utilization or mobilization, given that the reduction and release of ferritin-bound iron, crucial for the production of RBCs, is flavin-dependent. There is also animal evidence to suggest that riboflavin deficiency can lead to reduced iron absorption or increased gastrointestinal iron losses through greater shedding of mucosal cells or reduction in the number of intestinal villi with reduced absorptive surface area [70]. In addition, it is likely that riboflavin deficiency is indirectly implicated in the development of anemia through reducing vitamin B-6 status, given the requirement for riboflavin (as FMN) for the generation of pyridoxal 5’-phosphate (PLP) in tissues and evidence from human studies that riboflavin is the limiting nutrient for maintaining normal vitamin B-6 status in humans [71].

##### Prevalence of deficiency

Emerging evidence indicates that riboflavin deficiency is much more widespread than generally perceived but typically goes undetected in most countries. It was recently estimated that >4 billion people worldwide do not consume enough riboflavin (55% of the global population), with South Asia and Eastern and Southern Africa reported to have the highest prevalence of dietary inadequacy [72]. Data on riboflavin status from biomarker assessment are extremely limited, with very few countries including riboflavin biomarkers in their national nutrition surveys. The very limited biomarker data that exist show that females before and during pregnancy are at particular risk of deficient riboflavin status [73], especially so in some low- and middle-income countries (LMICs) [[74], [75], [76]]. Nationally representative data from the United Kingdom reported that 67% of teenage girls (11‒18 y) and 58% of females (19‒64 y) had a suboptimal or deficient riboflavin status [77]. In a convenience sample of reproductive-aged females in Canada, the prevalence of riboflavin deficiency was found to be 40% [78].

##### Key evidence for the role of riboflavin in nutritional anemia

Emerging evidence indicates that even marginal riboflavin deficiency contributes to nutritional anemia [69]. One study in >700 Chinese females showed that inadequate dietary riboflavin intakes were associated with an increased risk of anemia after 5 y of follow-up [79]. In a recent study of nonpregnant females from The Lao People’s Democratic Republic (*n* = 400), riboflavin status was found to be suboptimal in 97% of females sampled, and lower status was associated with an increased risk of anemia [80]. Likewise, in reproductive-aged females in Canada and Malaysia, a deficient status of riboflavin was associated with lower hemoglobin concentrations and a 2-fold greater risk of anemia [73]. Preliminary findings from a study of >2000 pregnancies in Ireland showed that riboflavin status in early pregnancy was a predictor of hemoglobin and the development of anemia in late pregnancy [69].

Evidence from RCTs, typically conducted in LMICs where clinical riboflavin deficiency is endemic, shows improved hematologic status in response to intervention with riboflavin, administered alone or in combination with other micronutrients. An RCT in Indonesian pregnant females found that the greatest improvements in hemoglobin concentrations were observed among those supplemented with riboflavin (5 mg/d) in combination with iron, compared with those supplemented with iron alone, IFA, or iron and vitamin A [81]. Similarly, additional improvements in hemoglobin and reduced prevalence of anemia were reported in pregnant females in rural China when riboflavin (1 mg/d) and vitamin A were administered in addition to IFA supplementation, compared with IFA only [82]. In contrast, in a large trial in >800 Cambodian females, the addition of multiple micronutrient supplementation (MMS) containing riboflavin (1.4 mg/d) did not confer significant additional benefit compared with iron-only supplementation for 12 wk, which resulted in increased hemoglobin concentrations [83].

Far fewer RCTs have been conducted in high-income countries (HICs) where riboflavin deficiency is less severe. A trial in young British females found that those with the lowest riboflavin status at baseline showed the greatest increase in hemoglobin in response to riboflavin supplementation (2 or 4 mg/d) [84]. This suggests that baseline riboflavin status may be a key factor in the extent of improvement in hematologic status with supplementation and that the beneficial effect may be confined to those with riboflavin deficiency. The differences in findings in the aforementioned randomized trials with regard to whether or not riboflavin intervention has beneficial effects on hemoglobin concentrations and risk of anemia are likely related to differences between trials in baseline riboflavin status of participants, riboflavin dose used, the duration of intervention, or the specific composition of the multiple micronutrient supplement administered (if any) along with riboflavin.

Riboflavin (as FAD or FMN) plays an essential role in interactions with other micronutrients, particularly vitamin B-6 and folate. The achievement of adequate vitamin B-6 status is dependent not only on dietary vitamin B-6 intake but also on riboflavin status. Notably, with a decline in riboflavin status, PLP concentrations were shown to decrease in a stepwise manner in a recent study of >5000 adults [71]. Also, riboflavin plays a particularly important role in maintaining one-carbon metabolism in individuals homozygous for the common C677T polymorphism in the gene encoding methylenetetrahydrofolate reductase (MTHFR) [85], raising the possibility that riboflavin requirements may be increased in individuals with the variant TT genotype in MTHFR, although this has not been proven to date. Furthermore, the adverse impact of riboflavin deficiency on vitamin B-6 status appears to be exacerbated in these individuals [71].

##### Gaps in current evidence and future research areas

The limited available data on the biomarker status of riboflavin indicate that females of reproductive age in LMICs across continents are at greatest risk of riboflavin deficiency [86]. Given the importance of riboflavin for both iron and folate metabolism, randomized trials are needed to investigate whether riboflavin intervention has a beneficial effect in alleviating anemia in pregnancy [69].

#### Zinc

##### Functional effects

Zinc is an essential trace mineral required by the body for numerous biological processes, and several mechanisms have been proposed that can potentially be related to anemia. First, in the heme biosynthesis pathway, the enzyme δ-aminolevulinic acid dehydratase is zinc-dependent, and studies in humans have shown that δ-aminolevulinic acid dehydratase activity is modified by dietary zinc intake [87,88]. Second, zinc deficiency may lead to increased erythrocyte fragility due to reduced antioxidant activity, as studies carried out in both animals and humans have shown that zinc restriction led to increased erythrocyte fragility [[89], [90], [91]]. A study conducted in zinc-deficient rats showed increased hemolysis, whereas zinc repletion rapidly restored hemolysis values to control levels [92]. Third, ≥4 zinc finger transcription factors are critical for erythroid cell proliferation and differentiation [93,94]; however, the extent to which these transcription factors are affected by dietary zinc remains to be determined. Fourth, decreased erythropoiesis is associated with low insulin-like growth factor-1 (IGF-1). A characteristic sign of zinc deficiency is stunting [95], which can be explained, partially, by a decreased IGF-1 concentration [96].

Preliminary observations also suggest other potential mechanisms of zinc deficiency and its role in anemia. Zinc supplementation provided to fish and rats with experimentally induced anemia showed a positive erythropoiesis response, highlighting a transferrin-zinc interaction [97,98]. In vitro results of erythropoiesis in bone marrow cells from anemic rats exposed to zinc supplementation led the authors to speculate that the effect may be mediated by increased autocrine secretion of EPO [98].

##### Prevalence of deficiency

Despite the several zinc status biomarkers commonly used, there is no single highly reliable indicator that allows one to unequivocally characterize the zinc status of an individual or group [99]. Thus, information on the global prevalence of zinc deficiency is challenging [100]; much of the data relies on plasma or serum zinc concentrations, which have inherent challenges of measurement and limitations of accuracy [101]. Gupta et al. [102] reported that 23 of 25 LMICs showed a prevalence of deficiency >20% in ≥1 population group, based on plasma zinc concentrations. Pooled data from 24 nationally representative surveys showed that the prevalence of zinc deficiency in both children and females was greater than that of iron deficiency, confirming the relevance of zinc deficiency globally. For nonpregnant females of reproductive age, prevalence of zinc deficiency was >20% in 13 of 15 datasets (>50% in Cambodia, Cameroon, Ecuador, Malawi, and Vietnam; no countries had <10% prevalence), whereas for preschool children, prevalence of zinc deficiency was >20% in 12 of 16 datasets (>50% in Cambodia, Cameroon, Malawi, and Vietnam; no countries had <10% prevalence) [18].

##### Key evidence for the role of zinc in nutritional anemia

Evidence from intervention trials has been difficult to ascertain as both zinc and iron deficiency are commonly concurrent, and comparisons across studies are difficult given the heterogeneity of intervention designs.

At least 12 observational studies (which include a wide variety of populations including children, adolescents, females of reproductive age, pregnant females, hospitalized adolescents, younger adults with eating disorders, and institutionalized older adults) have reported associations between zinc deficiency and anemia [[103], [104], [105], [106], [107], [108], [109], [110], [111], [112], [113], [114]], but only 8 of these reported some measurement of risk (as odd ratios) [[103], [104], [105], [106], [107],^109^,^111^,^113^]. Anemia also ranged widely across populations, from ∼5% to 46%, and zinc deficiency ranged from ∼7% to 72%. Greffeuille et al. [108] analyzed data from the global Biomarkers Reflecting Inflammation and Nutritional Determinants of Anemia (BRINDA) project and found that zinc deficiency was significantly associated with anemia in 5 of 13 available datasets of preschool children (Afghanistan, 24.1% zinc deficiency; Cambodia, 68%; Cameroon, 79.6%; Ecuador, 27.7%; and Mongolia, 78.4%) and in 4 of 12 datasets of females of reproductive age (Bangladesh, 54.4% zinc deficiency; Ecuador, 56.9%; Malawi, 63.4%; and Vietnam, 66.4%).

In trials that have investigated the effects of zinc supplementation (using zinc alone or zinc plus other micronutrients) on anemia and/or hemoglobin concentrations, data from studies among children are consistent with a lack of observed beneficial effects of zinc supplementation, whereas the findings varied among adults (Supplementary Table 5). This conclusion is also supported by 3 published reviews and meta-analyses [[115], [116], [117]] (Supplementary Table 6). Despite the aforementioned proposed mechanisms, most of the available evidence indicates that zinc interventions do not lead to improvements in hemoglobin concentration. Consequently, the relationship between zinc status and/or zinc supplementation and anemia remains unclear and warrants further investigation.

##### Gaps in current evidence and future research areas

One key challenge is that literature examining zinc supplementation alone (without iron) is scarce, thus making it difficult to isolate the effects of zinc from those of iron. Further, assessment of accurate zinc status is an inherent problem, which makes it difficult to assess in intervention studies. To elucidate a causal association between zinc and anemia, it would be ideal to conduct intervention studies that include zinc supplementation alone to confirm the potential mechanisms elucidating hemoglobin and/or RBC synthesis in a variety of zinc-deficient and replete populations.

#### Vitamin B-6

##### Functional effects

Vitamin B-6 and its derivatives (pyridoxine, pyridoxal, and pyridoxamine) are converted to PLP, the biologically active coenzyme and major circulating vitamin form in plasma. The most relevant mechanism linking vitamin B-6 deficiency with anemia is the impairment of hemoglobin synthesis, thus leading to hypochromic, microcytic anemia [118]. Specifically, PLP functions as a cofactor for δ-aminolevulinate synthase in erythrocytes, a crucial enzyme that catalyzes the rate-limiting step of heme biosynthesis in humans [119].

##### Prevalence of deficiency

The global prevalence of vitamin B-6 deficiency is unclear because biomarkers of status are rarely measured in population-based nutrition surveys. Although clinical vitamin B-6 deficiency very rarely occurs in HICs, given the widespread distribution of vitamin B-6 in most foods [120], substantial proportions are found to have functional deficiency (typically identified as a serum PLP value <20 nmol/L or <30 nmol/L) without classical deficiency signs [121]. Pregnant females and older people are considered to be at greatest risk of low vitamin B-6 status. In a large recent report, the first to provide global estimates of inadequate micronutrient intakes, it was estimated that 51% of the global population does not consume enough vitamin B-6 [72]. In the general population, vulnerable subgroups at risk of low vitamin B-6 status are pregnant females and older people.

##### Key evidence for the role of vitamin B-6 in nutritional anemia

Given that vitamin B-6 is required for heme synthesis, it is highly plausible that deficiency would contribute to IDA; however, evidence from human studies is somewhat limited.

Over the course of pregnancy, concentrations of PLP are reported to fall sharply [122]. There is some evidence implicating functional vitamin B-6 deficiency in pregnancy with an increased risk of anemia. Of note, a prospective study of Japanese pregnant females who were anemic and vitamin B-6–deficient at baseline demonstrated improved hemoglobin concentrations with administration of vitamin B-6 [123]. In Egyptian pregnant females with anemia, vitamin B-6 supplementation in combination with iron over a 3-wk intervention resulted in a greater increase in hemoglobin, compared with iron therapy alone, albeit the latter trial was nonrandomized [124].

Notably, riboflavin is essential for vitamin B-6 metabolism and plays a crucial role in generating PLP in tissues and PLP-dependent reactions [125]. Notably, in humans, consistent with its role in vitamin B-6 metabolism, a small intervention trial showed that riboflavin supplementation resulted in not only improved riboflavin status but also increased plasma PLP in older adults with low status of either vitamin at baseline [126]. The statuses of both nutrients are found to be highly correlated among adult populations [71,127]. The metabolic dependency of PLP on riboflavin is perhaps most evident in pregnancy, with emerging evidence that riboflavin may be the limiting nutrient for maintaining adequate vitamin B-6 status to cover increased needs during pregnancy [69,71].

##### Gaps in current evidence and future research areas

Future research is needed to increase the strength of the current evidence and elucidate a causal role of vitamin B-6 deficiency in the development of nutritional anemia. Clinical vitamin B-6 deficiency is uncommon, but further research is required to better understand functional deficiency, which occurs frequently, even in HICs, and is associated with increased risk of chronic diseases. An emerging possible mechanism for the role of vitamin B-6 in anemia is the mobilization of vitamin B-6 to sites of inflammation, where it may serve as a cofactor in pathways producing metabolites with immunomodulating effects [128]. Given that inflammation is implicated in many chronic diseases of aging, the relationship of vitamin B-6 with immune function and inflammation requires further investigation.

Functional vitamin B-6 deficiency is a particular concern in pregnancy, where it is associated with an increased risk of anemia and potentially a range of adverse outcomes for both mother and infant. Emerging evidence from human studies suggests that riboflavin may be a rate-limiting nutrient for the maintenance of normal plasma PLP concentrations across the lifecycle, with implications for heme biosynthesis and thus risk of anemia. Randomized trials are required to better understand the metabolic interaction between vitamin B-6 and riboflavin and the related health implications in order to support evidence-based dietary recommendations for both nutrients, particularly in the context of preventing anemia during pregnancy.

#### Vitamin C

##### Functional effects

Vitamin C (ascorbic acid), of importance to anemia, enhances the absorption of nonheme iron [[129], [130], [131], [132]] and stimulates iron uptake through the transferrin-iron uptake pathway [133]. Additionally, it inhibits ferritin autophagy [133,134] and plays a role in erythropoiesis by regulating EPO receptors [135] and promoting erythroid differentiation [136]. Folate, vitamin B-12, and vitamin C are also critical in erythropoiesis and in preventing megaloblastic anemia [137]. Although folate and vitamin B-12 help in the proliferation of erythroid cells, vitamin C prevents folate oxidation, enhancing its bioavailability [137,138].

##### Prevalence of deficiency

In HICs (across Europe, North America, and Asia Pacific), vitamin C deficiency in adults (defined as <11 *μ*mol/L in plasma) has ranged from 1% to 20%, with deficiency varying between ∼1% and 14% in females and ∼2% and 26% in males [139]. Studies in LMICs (Africa, Central America, South America, and Asia) revealed wider and higher rates of deficiency, with rates ≤79% in some populations [139]. Vitamin C deficiency in LMICs also varied by age, sex, and life stage, where deficiency in nonpregnant females ranged from ∼13% to 71% and in pregnant females ranged from ∼6% to 70% [139].

##### Key evidence for the role of vitamin C in nutritional anemia

In a recent systematic review and meta-analysis comparing hematologic outcomes in patients with IDA taking iron with vitamin C (cointervention) with those taking iron alone, the results suggested slight but significant increases in serum hemoglobin and ferritin concentrations in the cointervention group compared with the iron-only group, although the authors noted that these changes were likely not clinically significant [140]. It should be noted that the evidence included was heterogeneous, and some of it was of very low quality [140]. A 2016 Cochrane review also found a minor improvement in hemoglobin and ferritin concentrations with vitamin C (or another intervention) and iron (co-intervention) compared with iron alone in nonpregnant females; however, the co-intervention had a higher reduction than iron alone on anemia reduction [141]. Lastly, another meta-analysis revealed no significant differences between the cointervention and iron alone in participants with IDA of all ages [142]. The variation in findings may be due to including participants of all ages with various degrees of severity of anemia and differences in vitamin C and iron dosages across studies.

An equivalence RCT examined vitamin C with iron (cointervention) compared with iron alone for 3 mo in 440 adults, mainly females with IDA [143]. Both interventions were equally effective, and side effects were comparable. Notably, the cointervention improved mean corpuscular volume, a measure of RBC size often reduced in anemia, but the study was limited as most participants had IDA from causes unrelated to low intake, such as menorrhagia or gastrointestinal bleeding. Furthermore, anemia recurred within a year, likely because of absorption and bleeding issues not addressed in the study. The authors also suggested that a treatment period longer than 3 mo may be necessary to see the expected changes in hemoglobin concentrations.

##### Gaps in current evidence and future research areas

A recent review of global vitamin C status and deficiency prevalence emphasized that the studies from LMICs are typically small and conducted in food-insecure areas, whereas the studies in HICs tend to be larger epidemiologic studies [139]. This highlights the need for high-quality epidemiological studies in LMICs, including national surveys, to better assess vitamin C deficiency. Further, assessing vitamin C status accurately is challenging as the gold standard method, HPLC, is expensive [139]. Portable, cost-effective assessment tools are needed for remote populations with limited resources. Furthermore, there is a need for better evidence that should focus on vulnerable life stages, such as pregnant or lactating females, and explore optimal vitamin C dosages to understand if it enhances iron absorption and erythropoiesis.

In summary, although the evidence behind vitamin C mechanisms is well documented and of high quality, the evidence from intervention trials is lacking. Emerging research suggests, with regard to preventing megaloblastic anemia, that a dose as high as 500 mg/d of vitamin C might be needed to prevent folate oxidation [138]. Lastly, rising global obesity rates [121] complicate micronutrient status, including vitamin C [144,145], which impacts anemia risk, underscoring the need for more research on obesity’s effects on micronutrient and anemia status.

#### Vitamin E

##### Functional effects

Vitamin E (α-tocopherol) is a fat-soluble vitamin that functions primarily as an antioxidant and has a unique role in maintaining membrane integrity by preventing lipid peroxidation of PUFAs and is a recognized antiferroptotic agent [125]. Inadequate α-tocopherol intakes or fat malabsorption syndromes are the primary causes of vitamin E deficiency. Given the preventative role of α-tocopherol in lipid peroxidation in membranes, including erythrocytes, its deficiency increases susceptibility to erythrocyte hemolysis, which can contribute to anemia [146].

##### Prevalence of deficiency

A 2015 review that included over 170 studies found that ∼80% of the populations studied globally have serum α-tocopherol concentrations ≥30 *μ*mol/L, a concentration that some would consider “functionally optimal” [147]. Another large modeling study estimated that 67% of the global population (over 5 billion people), primarily those in the Americas, Middle East, and North Africa, have an insufficient dietary intake of α-tocopherol (from food sources, excluding supplements) [72,147].

##### Key evidence for the role of vitamin E in nutritional anemia

Animal studies first observed α-tocopherol deficiency and anemia as a symptom [148]. In human studies, vitamin E supplementation has been shown to normalize the shortened RBC lifespan in children with cystic fibrosis [149] and has shown that anemia in newborn infants was responsive to α-tocopherol supplementation [150,151].

Various studies have reported on anemia that is responsive to vitamin E supplementation in malnourished populations with mixed findings. In rural Bangladesh, among pregnant females with minimal iron deficiency, plasma α-tocopherol concentrations were associated with hemoglobin concentrations, suggesting that the anemia may be responsive to vitamin E [112]. Moreover, in a placebo-controlled trial of adults with mild anemia in Pakistan, vitamin E supplementation for 3 mo was associated with improved hemoglobin concentrations; thus, the authors suggest that vitamin E supplementation may offer a simple and inexpensive mode of treatment in mild anemia [152]. In contrast, another study of vitamin E supplementation in transfusion-dependent children with anemia (6–18 y, *n* = 30 with β-thalassemia, *n* = 30 with sickle cell disease) showed that serum concentrations of both α-tocopherol and selenium were depleted relative to the healthy control population; however, a combination of vitamin E and vitamin C supplements in such patients did not improve the anemia [153]. However, findings are not consistent between studies in patients with sickle cell anemia [154], and the sample size in these studies was small.

Vitamin E may be an important nutrient to decrease the incidence of anemia; however, vitamin E deficiency status is difficult to assess [155]. For example, in a study in Vietnamese children given a daily multivitamin-mineral supplement, only plasma α-tocopherol concentrations increased significantly [156]. However, after 6 mo of supplementation, anemia still affected a quarter of infants, and there was no iron deficiency, suggesting that multiple factors were contributing to the anemia [156]. Similarly, Allen et al. [157] reported that 70% of rural Mexican children aged 18 to 36 mo (*n* = 219) had low hemoglobin (<115 g/L), 60% had low hematocrit, 48% were ferritin-deficient, and 70% had deficient plasma α-tocopherol concentrations. However, the supplement provided did not contain vitamin E, suggesting that α-tocopherol is often overlooked when considering the causes of anemia.

##### Gaps in current evidence and future research areas

Challenges remain in obtaining accurate global estimations of vitamin E status, as there is a lack of scientific consensus on thresholds for its deficiency. Furthermore, biomarkers of human α-tocopherol status are not commonly measured and are dependent on a variety of factors, and measures of dietary assessments are poorly correlated with α-tocopherol status.

Some disorders may predispose patients to vitamin E deficiency–dependent anemia, including inflammatory bowel disease [158]. Often, metabolic syndrome is seen as a condition of overnutrition, but a study from Ethiopia reported that 25% of 324 patients with this condition admitted to the hospital were anemic [159]. Furthermore, persons with metabolic syndrome have shown increased inflammation and impaired vitamin E bioavailability [160]; thus, anemia in certain conditions should be investigated further.

There is emerging evidence for the key role of α-tocopherol in the prevention of anemia and its likely role as an antioxidant protecting the red cell membrane in circulation, but the precise mechanism(s) of its actions remains under investigation. Studies of the production of erythrocytes in bone marrow have revealed the complex role of the mitochondria, the accumulation of iron, and the involvement of oxidative stress [161,162]. Recent evidence suggests that α-tocopherol also has a role in increasing erythrocyte production, possibly by protecting bone marrow stem cells and erythroid progenitor cells from ferroptosis and may facilitate erythrocyte production by modulating oxidative damage [151,163].

### Potential/emerging evidence (vitamin D and copper)

#### Vitamin D

##### Functional effects

Vitamin D is best known for its role in calcium regulation and bone health [164]. However, many cells, including those involved in erythropoiesis and the immune system, have vitamin D receptors, indicating additional roles for vitamin D beyond calcium homeostasis [164]. Both dietary and synthesized vitamin D (through sun exposure) are converted into 25-hydroxyvitamin D [25(OH)D], the most accepted biomarker of vitamin D status [164,165].

##### Prevalence of deficiency

The global prevalence of vitamin D deficiency varies significantly depending on the cutoff used to define low 25(OH)D concentrations. A recent global survey reported that ∼16% of individuals have concentrations below 30 nmol/L (severe), 50% below 50 nmol/L (moderate), and 77% below 75 nmol/L (mild) [166]. However, there is ongoing controversy over what constitutes an adequate 25(OH)D concentration, with recommendations ranging from 50 nmol/L to 100 nmol/L depending on health goals and assay accuracy.

##### Key evidence for the role of vitamin D in nutritional anemia

**FIGURE 2:**
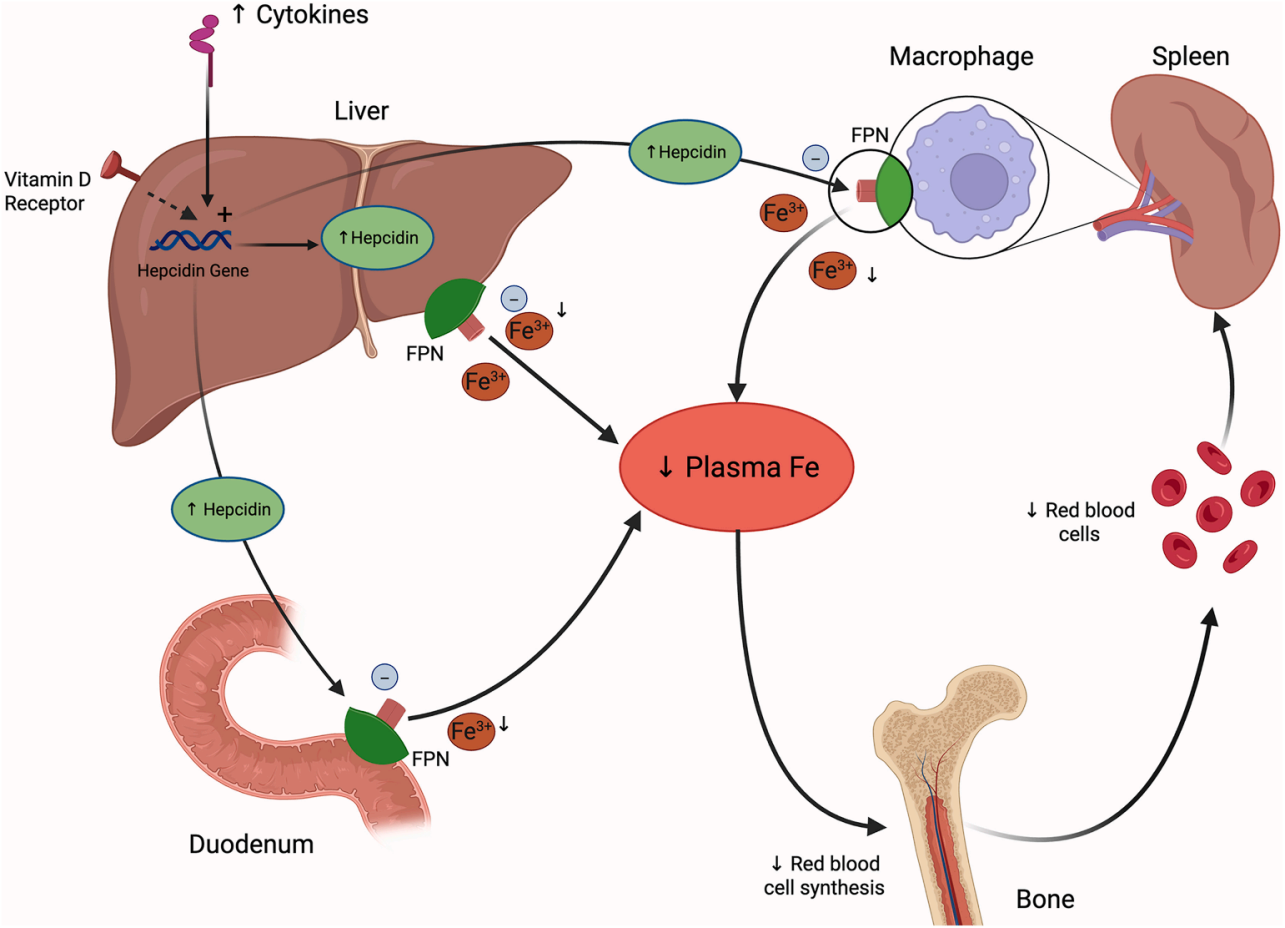
Anemia of inflammation as a hypothesized result of vitamin D deficiency. Anemia of inflammation is the second leading cause of anemia after iron (Fe) deficiency [2]. During inflammation, proinflammatory cytokines (e.g., IL-6) directly inhibit erythropoiesis and increase the synthesis of hepcidin [167]. Hepcidin binds to ferroportin (FPN), the sole cellular transmembrane Fe exporter on the plasma membranes of enterocytes, macrophages, and hepatocytes [2], blocking Fe release into circulation and making it unavailable for erythropoiesis. The prevailing theory of the potential mechanism by which vitamin D modifies anemia of inflammation is that vitamin D, in its active form of 1,25-dihydroxyvitamin D, may suppress cytokine production and bind to a vitamin D–responsive element in the hepcidin gene, downregulating hepcidin transcription. This may reduce hepcidin concentration and increase cellular Fe export, allowing Fe to be released from enterocytes, macrophages, and hepatocytes, and become available for erythropoiesis [168]. Figure reproduced with permission from Springer Nature and modified in BioRender. T.J. Green (2026; https://BioRender.com/moen571).

Although several mechanisms have been proposed to explain how vitamin D may be involved in anemia, the prevailing theory suggests that vitamin D helps protect against anemia by reducing inflammation. Figure 2 [2,167,168] (reproduced with permission from Springer Nature [1]) illustrates anemia of inflammation, highlighting how inflammation can alter iron metabolism and erythropoiesis, leading to reduced hemoglobin synthesis despite adequate iron stores.

Observational studies demonstrate an inverse relationship between 25(OH)D and anemia [169,[170], [171], [172], [173], [174], [175]]. For instance, data from the United States NHANES (2001‒2006) indicate that individuals aged >17 y with 25(OH)D concentrations below 50 nmol/L are 1.6 times more likely to be anemic than those with higher concentrations [170]. Although 25(OH)D is generally associated with hemoglobin concentrations, in most studies in which it was reported, the effect size was relatively small [170,176,177]. Most studies also suggest a threshold effect, with no further associations observed at concentrations typically >50 nmol/L [169,170,172,174].

The association between 25(OH)D and iron deficiency, IDA, and biomarkers of iron has not been well studied because the complex relationship between iron and inflammation biomarkers challenges the interpretation of studies. For example, ferritin was not associated with 25(OH)D in most studies, but ferritin can be increased in the presence of inflammation despite poor iron status. If vitamin D acts as an immunomodulator, lower serum concentrations of 25(OH)D should correlate with higher concentrations of hepcidin and proinflammatory cytokines. In a small study of hospitalized children (*n* = 32), serum 25(OH)D was lower, and IL-6 and hepcidin concentrations were higher in the children with anemia having acute infection [178]. In a large study of older Mexican adults (*n* = 783, >60 y), no association was found between 25(OH)D and hepcidin [179]. Although anemia prevalence was high at 36% in the study population, <10% of participants had a 25(OH)D concentration below 50 nmol/L.

To the best of our knowledge, 10 RCTs to date have examined the effects of vitamin D supplementation on anemia or related biomarkers, which have shown inconsistent results [[180], [181], [182], [183], [184], [185], [186], [187], [188], [189]] (Supplementary Table 7). Most studies found higher 25(OH)D concentrations at follow-up in the vitamin D group compared with placebo; however, none of the studies reported that vitamin D lowered rates of anemia relative to placebo. In RCTs assessing hemoglobin concentrations, 2 trials reported higher hemoglobin concentrations in participants receiving vitamin D supplementation compared with placebo [181,188], whereas 4 trials found no difference [183,184,186,189].

In 1 study, females with marginal iron deficiency (*n* = 50) consumed iron-fortified cereal (containing 9 mg of elemental iron) for 8 wk and were given either 38 *μ*g/d of vitamin D or a placebo [181]. The females who received vitamin D had a 7 g/L higher hemoglobin concentration. In contrast, in a study of individuals in Norway (*n* = 251), participants randomly assigned to either 10 or 25 *μ*g/d vitamin D groups did not have a higher mean hemoglobin concentration than that in the placebo group after 16 wk of supplementation [186].

In a study of pregnant females in Bangladesh, prenatal vitamin D supplementation did not influence maternal biomarkers of iron transport, regulation, or iron-deficiency erythropoiesis [180]. Among females with vitamin D deficiency [25(OH)D <30 nmol/L] before the intervention, an observed negative effect of prenatal high-dose vitamin D supplementation on serum ferritin at delivery may reflect an anti-inflammatory effect of vitamin D [180].

Several small trials have examined the effect of vitamin D on iron biomarkers. In the 6 trials that included ferritin measurements, no significant differences were found between the vitamin D groups and placebo groups [181,182,[185], [186], [187],^189^]. In 1 study, plasma hepcidin was lower in healthy adults (*n =* 28) randomly assigned to a single bolus of vitamin D3 (6250 *μ*g/d) than in the placebo group after 1 wk [187]. Not unexpectedly, during the short follow-up period, hemoglobin did not differ between the groups. None of the other studies reported an effect of vitamin D supplementation on hepcidin.

##### Gaps in current evidence and future research areas

Current evidence from observational studies and RCTs is insufficient to establish a clear causal role for vitamin D in the development of anemia. Observational studies show weak associations, and the small effect size is complicated by the interactions among vitamin D, inflammation, iron biomarkers, and anemia. RCTs have been underpowered and often involve populations with low rates of anemia and vitamin D deficiency. Well-designed studies with populations facing high rates of anemia and vitamin D deficiency are needed to better assess the potential benefits of vitamin D.

#### Copper

##### Functional effects

Copper acts as a redox catalyst in various metabolic reactions and exists in biological systems in 2 oxidation states, Cu^2+^ (or cupric copper) and Cu^1+^ (or cuprous copper) [190]. Because of its high redox potential and reactivity, excess cellular copper is toxic, possibly causing oxidative stress and leading to cuproptosis, a copper-related form of programmed cell death [191]. Copper-dependent enzymes, called cuproenzymes, are vital in a variety of biological processes, including hematopoiesis and iron metabolism [192]. Most serum copper is contained within the liver-derived, circulating multicopper ferroxidase ceruloplasmin (CP), which plays an important role in iron metabolism.

In humans, copper deficiency is typically defined by low serum copper (70‒80 *μ*g/dL or below), low serum CP (varies by sex, oral contraceptive use, inflammation, and aging; deficiency is generally <20 mg/dL), and low 24-h urinary copper [193]. Low serum copper concentration is a specific bioindicator of copper status, but paradoxically, it can be in the normal range even when some copper-dependent enzyme functions are impaired, indicating a potential latent deficiency [194]. Efforts to identify biomarkers of covert (or sub-clinical) copper deficiency have been largely unsuccessful to date [195,196]. Collectively, these observations highlight challenges in defining an ideal biomarker of copper nutriture and in identifying individuals at risk of copper deficiency.

##### Prevalence of deficiency

At least one-fourth of United States adults consume less than the Estimated Average Requirement (EAR) for copper; however, establishing recommended dietary intakes for copper is complex given the difficulty in measuring copper intake and the lack of robust biomarkers, which hinder the ability to measure the potential pathological outcomes of copper depletion [195,[197], [198], [199]]. Moreover, it has been suggested that the current United States recommended dietary allowances (RDAs) for copper may be low, as RDAs in the United Kingdom and Australia are notably higher [200,201]. Copper depletion can be ≥20% in specific population subsets, such as adults receiving total parenteral nutrition [202], bariatric surgery patients [203], and individuals with liver cirrhosis [204]. Copper deficiency has also been described in nephrotic syndrome, celiac disease, and rare genetic disorders such as Menkes disease and secondarily in a number of conditions including malnutrition, anorexia nervosa, and Crohn disease [205,206]. Moreover, geographic factors may influence copper consumption and thus the prevalence of deficiency [207].

##### Key evidence for the role of copper in nutritional anemia

Copper deficiency has been commonly associated with impaired hematopoiesis, with the most frequent outcomes being anemia, leukopenia, and neutropenia [208]. Interestingly, both low and high serum copper concentrations were associated with unexplained anemia in NHANES II, indicating a possible U-shaped response curve [209]. Copper-deficiency–related anemia may present as macrocytic, normocytic, or microcytic [210]. Hematologic outcomes of copper deficiency may mimic myelodysplastic anemia [211].

**FIGURE 3:**
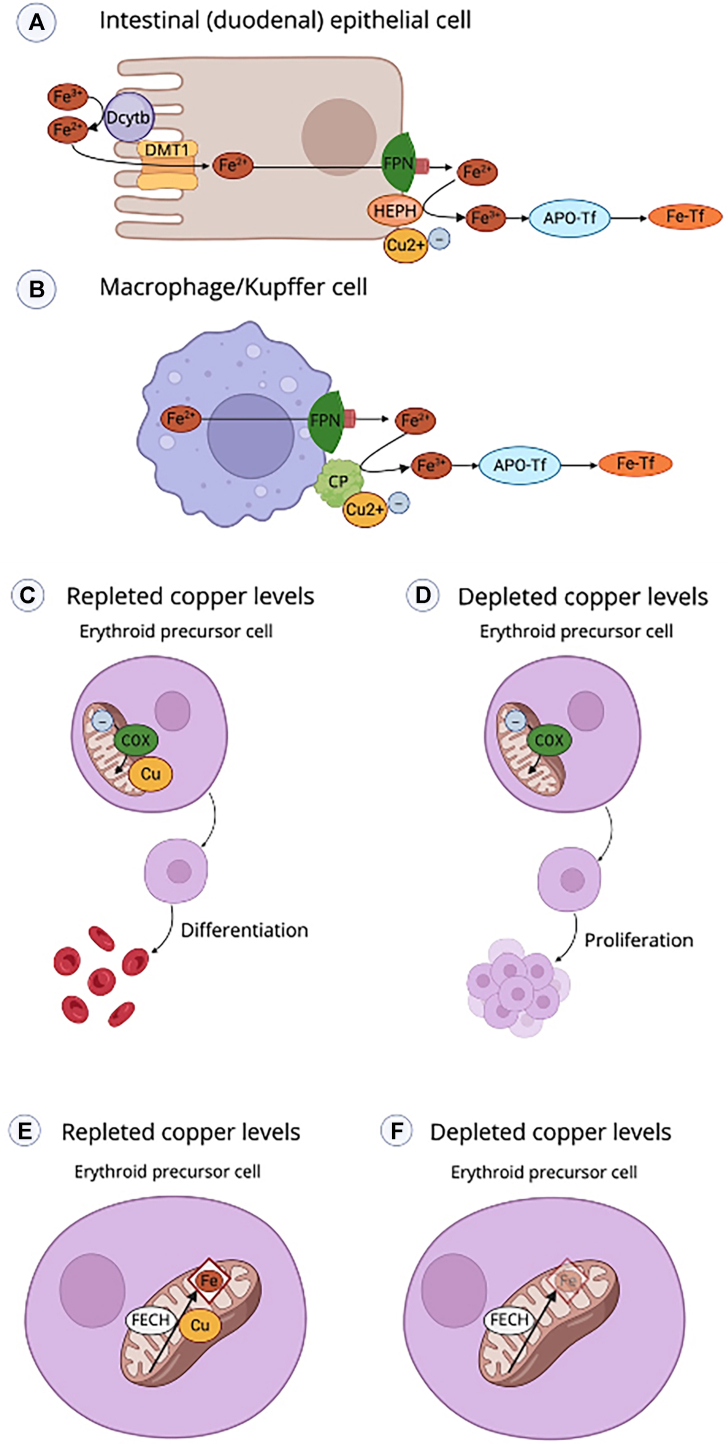
The hypothesized role of copper (Cu) in the development of anemia. (A) Once iron (Fe, in the form Fe^2+^) is exported from the basolateral membrane of enterocytes by the transporter ferroportin (FPN) in the duodenal epithelium, it becomes oxidized (to the form Fe^3+^) by the Cu-dependent ferroxidase enzyme, hephaestin (HEPH). Apotransferrin (APO-Tf) binds to Fe^3+^, forming the molecule holotransferrin (Fe-Tf), allowing Fe to be transported through the bloodstream. (B) Once Fe (in the form Fe^2+^) is exported from the basolateral membrane of macrophages or Kupffer cells in the liver, Fe becomes oxidized (to the form Fe^3+^) by the Cu-dependent ferroxidase enzyme CP. APO-Tf binds to Fe^3+^, forming the molecule Fe-Tf, allowing Fe to be transported through the bloodstream. Low Cu oxidase activity impairs Fe export and integration into Fe-Tf, decreasing available Fe^3+^ for erythropoiesis. (C) In the mitochondria of erythroid precursor cells, the Cu-containing enzyme cytochrome c oxidase (COX) is the final electron acceptor in the mitochondrial electron transport chain required for ATP production, and thus cell differentiation, and specifically RBC production. (D) Low Cu concentrations impair COX function, leading to impaired electron transport function and decreased cell differentiation/increased cell proliferation, therefore reducing the number of fully functional, mature RBCs. (E) In the mitochondria of erythroid precursor cells, Cu interacts with the terminal enzyme in the heme biosynthetic pathway, ferrochelatase (FECH), which catalyzes the insertion of an Fe atom into the protoporphyrin ring to form heme. (F) Depleted Cu concentrations may reduce the activity of FECH, potentially leading to low heme biosynthesis in erythroid precursors. CP, ceruloplasmin; Dcytb, duodenal cytochrome b; DMT1, divalent metal transporter 1; RBC, red blood cell. Created in BioRender. C.C. Farrell (2026; https://BioRender.com/26w4ujo).

The mechanism(s) by which copper depletion causes anemia has not been firmly established [208], although 3 logical hypotheses have been put forth (Table 1 and Figure 3). First, copper depletion may negatively affect the activity of 2 cuproenzymes directly involved in iron metabolism, hephaestin (HEPH) and CP [192]. Low activity of HEPH and CP may impair intestinal iron absorption and reduce iron release from stores, respectively, leading to hypoferremia, iron-restricted erythropoiesis, and anemia. Second, low copper impairs mitochondrial function in erythroid progenitor cells, which decreases differentiation, thus reducing the number of fully functional, mature RBCs [212]. Third, low intracellular copper may impair heme production in developing RBCs, thus leading to low heme concentrations in erythrocytes and subsequently anemia. Although the mechanism has not been clarified, it could be that low copper reduces the enzyme activity of ferrochelatase [213], which catalyzes the insertion of iron in the final step in the heme biosynthetic pathway.

##### Gaps in current evidence and future research areas

Low copper intake in the general population may be more common than generally perceived, and most experimental copper depletion studies were carried out only in males. Clinical features of copper deficiency can be seemingly nonspecific, impacting several organ systems [214]. Diagnosis of copper deficiency is complicated given the lack of specific and sensitive biomarkers of copper nutritional status [214]. Low serum copper and CP are established bioindicators of moderate-to-severe copper deficiency that can be corrected with copper supplementation [208]; however, other (patho)physiologic changes may also impact serum copper and CP, such as chronic inflammation and pregnancy. Many studies have assayed other cuproenzymes in blood and attempted to relate outcomes to copper nutritional status [195,199]; however, the activity of these proteins did not robustly respond to copper supplementation. Cuproenzymes studied include erythrocyte and leukocyte superoxide dismutase, platelet cytochrome C oxidase, and plasma diamine oxidase [215,216]. Identification of new bioindicators of copper status is thus imperative. Furthermore, although the molecular mechanisms by which copper deficiency causes anemia are unclear, they may involve perturbed iron metabolism, mitochondrial dysfunction, and/or impaired heme biosynthesis in erythrocyte precursors. Clarifying the molecular mechanisms underlying copper-deficiency anemia may accelerate diagnostic capabilities and lead to the development of new approaches to more rapidly and effectively identify individuals at increased risk of copper deficiency.

### Non-nutritional causes of anemia

Although nutritional deficiencies are a major contributor to anemia, a substantial proportion of cases arise from non-nutritional causes, including chronic inflammation [2], disease or infection [3], genetic hemoglobin disorders (e.g., sickle cell) [4], blood loss, and/or heavy menstrual bleeding. Understanding the etiology of anemia is critical because it allows for targeted interventions that address the specific micronutrient and/or cause of the anemia. This is especially critical in settings with scarce resources because it allows programs to direct limited funding, supplies, and personnel toward interventions that will be most effective.

### Holistic approaches to treat, prevent, and reduce anemia globally

A global anemia strategy is most effective when it combines targeted nutrition-specific interventions, infection and disease control, health systems integration, policy support, and research-informed innovation, rather than relying on iron supplementation alone. This strategy goes beyond the single-nutrient approach to address the multifactorial causes of anemia. MMS in pregnancy is increasingly being adopted and scaled up in many low-resource countries as a holistic nutritional intervention that simultaneously provides 15 different micronutrients (targeting deficiencies beyond IFA alone) and has been shown to improve birth outcomes with comparable effects on maternal anemia prevention, despite containing half the amount of iron as that of standard IFA [[217], [218], [219]]. Integrating MMS into routine antenatal care and health system infrastructure—including supply chains, monitoring, and policy frameworks—enhances coverage, adherence, and impact, exemplifying how holistic nutritional interventions can be operationalized at scale [220].

In addition to supplementation, food fortification (including biofortification) and dietary diversification strategies offer holistic approaches to addressing multiple nutrient gaps simultaneously. Fortifying staple foods with iron, folate, vitamin A, and other micronutrients offers a means to improve population-level nutrient intake without requiring major dietary or behavioral change. Further, promoting dietary diversity—through increased consumption of legumes, animal-source foods, and nutrient-rich fruits and vegetables—supports broader micronutrient adequacy and overall health beyond a single deficiency.

We acknowledge some limitations of our work. A formal and structured protocol to review the existing literature was not employed; rather, each author exercised individual judgment in identifying and summarizing the most relevant and rigorous data to be included in this work. Second, there is considerable heterogeneity across the included studies (e.g., study design, population, and geographic setting), which may limit direct comparability across studies and generalizability beyond each study. Lastly, we acknowledge that nutritional anemia is often caused by >1 single micronutrient deficiency and that the relationship is often confounded or biased by various concurrent nutritional and non-nutritional factors (e.g., socioeconomic factors, inflammation, and disease state).

In conclusion, holistic and tailored approaches to treat, prevent, and reduce anemia should be employed while promoting the micronutrients with the strongest evidence for the prevention and treatment of anemia and focusing new research on those nutrients with less and/or emerging evidence.

## Author contributions

The authors’ responsibilities were as follows– CDK, CCF, ADG: designed the research; CCF, JLB, JFC, ADG, TJG, LH, MCK, HM, AFP, MR, MGT, CDK: contributed to content and wrote the paper; CDK: takes primary responsibility for content; and all authors: read and approved the final manuscript.

## Declaration of Generative AI and AI-assisted technologies in the writing process

The authors declare that no generative AI or AI-assisted technologies were used in the writing of this manuscript.

## Funding

dsm-firmenich provided funding for publication fees and author honorariums; however, it had no role in the writing or interpretation of the data.

## Conflict of interest

The authors report no conflicts of interest.
