# Supplementary material for: The Role of Micronutrient Deficiencies in Nutritional Anemia Beyond Iron: An Expert Perspective on the Scope and Strength of the Current Evidence
Source: Adv Nutr. 2026 Jul 3;17(9):100697. doi: 10.1016/j.advnut.2026.100697 (PMC13486352; doi:10.1016/j.advnut.2026.100697)
Supplement: Supplementary file 1 [file mmc1.docx]

**Supplementary Online Content**

**Supplementary Table 1:** Global prevalence of folate deficiency based on serum folate concentrations

| **Author, year** | **Country** | **Population** | ***n*** | **Supplement users included** | **Method** | **Cut-off for deficiency** | **Prevalence of deficiency (%)** |
| --- | --- | --- | --- | --- | --- | --- | --- |
| **Mandatory food fortification:** | | | | | | | |
| Barnabé et al. 2015 [1] | Brazil | Children (≤6y)  Older adults (≥60y)  Pregnant women  Lactating women  All groups (0.5-91y) | 103  262  291  54  710 | Y | Chemiluminescence immunoassay | <10nmol/L | 0  <1  <1  0  <1 |
| Palchetti et al. 2022 [2] | Brazil | Adults (20-59y)  Older adults (≥60y)  All adults (≥20y) | 302  308  610 | Y | Chemiluminescence  immunoassay | <10nmol/L | 2  <1  2 |
| Zhou et al. 2023 [3] | US  (NHANES, 2007-18) | Adults (≥19y)  Non-Hispanic White  Non-Hispanic Black  Non-Hispanic Asian  Hispanic | 31,128  12,891  6,725  2,991  7,966 | Y | Microbiological assay (2007-10)  LC-MS/MS (2011-18) | <7nmol/L | <1  <1  <1  <1  <1 |
| **Voluntary food fortification:** | | | | | | | |
| Jones et al. 2023 [4] | UK  (NDNS,  2016-19) | Children (4-10y)  Adolescents (11-18y)  Adults (19-64y)  Older adults (≥65y) | 142  230  727  221 | Y | LC-MS/MS | <7nmol/L | 1  9  11  7 |
| **No food fortification:** | | | | | | | |
| Solvik et al. 2020 [5] | Norway | Children (4-6y) | 197 | Y | Microbiological assay | <6.8nmol/L  <10nmol/L  <13.4nmol/L | 3  13  37 |
| Sundarakumar et al. 2021 [6] | India | Adults (≥45y) | 1640 | N | Chemiluminescence  immunoassay | <6.8nmol/L | 11 |

NDNS, National Diet and Nutrition Survey; NHANES, National Health and Nutrition Examination Survey; LC-MS/MS, liquid chromatography coupled to tandem Mass spectrometry

**Supplementary Table 2:** Summary of trials assessing the effect of folic acid intervention on hematological parameters

| **Author, year** | **Country** | **Population** | **Intervention** | **Intervention duration** | ***n*** | **Hemoglobin pre-intervention (g/dL)** | ***n*** | **Hemoglobin post-intervention (g/dL)** | **Main findings relevant to folic acid on hematological parameters** |  |
| --- | --- | --- | --- | --- | --- | --- | --- | --- | --- | --- |
| Mara et al. 2001 [7] | Czech Republic | Females (4 days post-partum) | No supplement (control) | Daily for 2 months | 20 | 10.7 (1.5) | 20 | 11.9 (0.8) | *Hb, Hct and erythrocyte count*: significant increase in both treatment groups, no significant increase in control group  *MCV, MCHC and MCH*: no difference between groups | |
|  |  |  | 80mg Fe +30mg ascorbic acid |  | 20 | 10.6 (1.3) | 20 | 12.4 (0.7) |  |  |
|  |  |  | 80mg Fe _+ 30mg ascorbic acid + 350µg FA |  | 20 | 10.3 (1.1) | 20 | 12.7 (0.6) |  |  |
| Juarez-Vazquez et al. 2002 [8] | Mexico | Pregnant women (14-27 GW) with IDA | 80mg Fe (control) | Daily for 2 months | 182 | 10.4 (0.9) | 132 | 11.2 (1.5) | *Hb and erythrocyte count*: significantly increase in FA group.  *Hct*: significant increase in both groups.  *MCV*: no difference in or between groups | |
|  |  |  | 80mg Fe + 370µg folinic acid |  | 189 | 10.2 (1.0) | 135 | 11.6 (1.7) |  |  |
| Christian et al. 2003 [9] | Nepal | Pregnant women (1^st^ trimester) |  | Daily until 3 months post-partum | *32 GW* |  | |  | *Hb and anemia*: no difference between control and FA group | |
|  |  |  | 1000µg vitamin A (control) |  | 283 | 11.6 (1.8) | 179 | -- |  |  |
|  |  |  | 1000µg vitamin A + 400µg FA |  | 204 | 11.5 (2.0) | 138 | -2.5 (-7.9, 2.9) ^†^ |  |  |
|  |  |  | 1000µg vitamin A + 400µg FA + 60mg Fe  *Two additional treatment arms included vitamin A+Fe+FA+Zn and vitamin A+Fe+FA+Zn+MMN* |  | 202 | 11.5 (1.8) | 136 | 13.8 (8.3, 19.2) ^†^ |  |  |
|  |  |  |  |  | *6 weeks post-partum* |  |  |  | *Hb and anemia*: no difference between control and group receiving FA alone. | |
|  |  |  | 1000µg vitamin A (control) |  | 236 | 11.2 (1.8) | 236 | -- |  |  |
|  |  |  | 1000µg vitamin A + 400µg FA |  | 175 | 11.3 (1.7) | 175 | 0.9 (-3.6, 5.3) ^†^ |  |  |
|  |  |  | 1000µg vitamin A + 400µg FA + 60mg Fe  *Two additional treatment arms included vitamin A+FE+FA+zinc and vitamin A+Fe+FA+zinc+MMN* |  | 178 | 12.2 (1.3) | 178 | 9.9 (5.5, 14.4) ^†^ |  |  |
| Hadler et al. 2008 [10] | Brazil | Children (6-24 months) | *Anemic:* | 5 days per  week for ~3 months |  |  |  |  | *Hb*: significant increase in both groups.  *Anemia*: significantly lower prevalence in FA group (14% FA *vs* 35% placebo) | |
|  |  |  | 4.2mg/kg Fe + placebo |  | 43 | 9.9 (0.7) | 43 | 11.3 (1.0) |  |  |
|  |  |  | 4.2mg/kg Fe + 50µg FA |  | 43 | 10.0 (0.8) | 43 | 11.5 (0.8) |  |  |
|  |  |  | *Non-anemic:* |  |  |  |  |  | *Hb:* significantly improved in FA group  *Anemia*: no difference in prevalence of anemia between groups (6% FA *vs* 10% placebo) | |
|  |  |  | 1.4mg/kg Fe + placebo |  | 39 | 11.8 (0.6) | 39 | 12.0 (0.9) |  |  |
|  |  |  | 1.4mg/kg Fe + 50µg FA |  | 36 | 11.7 (0.6) | 36 | 12.1 (0.8) |  |  |
| Rosado et al. 2010 [11] | Mexico | Children (6-42 months) | 20mg Fe | Daily for 4 months | 40 | 10.5 (10.2, 10.7) | 40 | 11.9 (11.6, 12.3) | *Hb*: significant increase in Fe and Fe+FA groups.  *Anemia:* reduced in Fe and Fe+FA groups | |
|  |  |  | 12.5mg Fe + 50µg FA  *Three additional treatment arms included a MN supplement, micronutrient fortified porridge powder, and Zn+Fe+ascorbic acid fortified water* |  | 42 | 10.7 (10.5, 11.0) | 42 | 12.2 (11.9, 12.3) |  |  |
| Kumar et al. 2017 [12] | India | Children (6-30 months) | Placebo | Daily for 6 months | 64 | 10.1 (1.3) | 63 | 9.9 (1.4) | *Hb*: no significant difference between placebo and FA group | |
|  |  |  | 150µg FA  *Two additional treatment arms included vitamin B12 and vitamin B12+FA* |  | 67 | 10.4 (1.5) | 66 | 9.7 (1.7) |  |  |

^†^ Relative difference compared to control presented as mean (95% CI).

Data presented as mean (SD) or mean (95% CI). FA, folic acid; Fe, iron; GW, gestational weeks; Hb, hemoglobin; Hct, hematocrit; IDA, iron deficiency anemia; MCHC, mean cell hemoglobin concentration; MCH, mean cell hemoglobin; MCV, mean cell volume; MMN, multiple micronutrients; Zn, zinc.

**Supplementary Table 3:** Global prevalence of vitamin B12 deficiency based on total vitamin B12 and methylmalonic acid (MMA) concentrations

| **Author, year** | **Country** | **Population** | ***n*** | **Supplement users included** | **Method** | **Cut-off for deficiency** | **Prevalence of deficiency (%)** |
| --- | --- | --- | --- | --- | --- | --- | --- |
| Den Elzen et al. 2008 [13] | The Netherlands | Older adults (≥85y) | 423 | N | Radioassay | <150pmol/L | 16 |
| Shoba et al. 2011 [14] | India | Older adults (60-70y)  Older adults (>70y)  All older adults (≥60y)  Vegetarian  Non-vegetarian  Older adults (60-70y)  Older adults (>70y)  All older adults (≥60y)  Vegetarian  Non-vegetarian | 136  38  175  99  76  136  38  175  99  76 | Y | Chemiluminescence immunoassay  GC-MS | <150pmol/L  *MMA*  >0.30 µmol/L | 18  9  16  19  12  56  53  55  62  46 |
| Samuel et al. 2013 [15] | India | Pregnant women  (18-40y) | 366 | N | Chemiluminescence immunoassay  GC-MS | <150 pmol/L  150-250 pmol/L  *MMA*  >0.26 µmol/L | 51  32  76 |
| Barnabé et al. 2015 [1] | Brazil | Young children (≤6y)  Older adults (≥60y)  Pregnant women  Lactating women  All groups (0.5-91y)  Young children (≤6y)  Older adults (≥60y)  Pregnant women  Lactating women  All groups (0.5-91y) | 103  262  291  54  710  103  262  291  54  710 | Y | Chemiluminescence immunoassay | <148 pmol/L  148-221pmol/L | 0  4  8  2  5  1  15  34  7  20 |
| Ramírez-Vélez et al. 2016 [16] | Colombia | Pregnant women  (13-49y) | 1781 | Y | Chemiluminescence  Immunoassay | <148pmol/L  148-221pmol/L | 19  41 |
| Chandyo et al. 2016 [17] | Nepal | WRA (13-35y) | 500 | N | Microbiological assay  GC-MS | <150pmol/L  *MMA*  >0.26µmol/L | 42  82 |
| Finkelstein et al. 2021 [18] | India | WRA (15-40y) | 978 | Y | Chemiluminescence  Immunoassay | <148pmol/L  <221pmol/L | 48  74 |
| Solvik et al. 2020 [5] | Norway | Children (4-6y) | 197 | Y | Microbiological assay  GC-MS/MS | <148pmol/L  148-221pmol/L  *MMA*  >0.26µmol/L | 0  <1  1 |
| Sundarakumar et al. 2021 [6] | India | Adults (≥45y) | 1640 | N | Chemiluminescence  immunoassay | <148pmol/L | 42 |
| Palchetti et al. 2022 [2] | Brazil | Adults (20-59y)  Older adults (≥60y)  All adults (≥20y) | 302  308  610 | Y | Chemiluminescence  immunoassay | <148pmol/L | 22  21  21 |
| Zhou et al. 2023 [3] | US  (NHANES, 2007-18) | Adults (≥19y)  Non-Hispanic White  Non-Hispanic Black  Non-Hispanic Asian  Hispanic  Adults (≥19y)  Non-Hispanic White  Non-Hispanic Black  Non-Hispanic Asian  Hispanic | 31,128  12,891  6,725  2,991  7,966 | Y | Chemiluminescence  immunoassay | <148pmol/L  <221pmol/L | 4  3  5  4  3  12  13  11  14  12 |

GC-MS, gas chromatography-mass spectrometry; MMA, methylmalonic acid; MS, mass-spectrometry; NHANES, National Health and Nutrition Examination Survey; WRA, women of reproductive age.

**Supplementary Table 4:** Summary of trials assessing the effect of vitamin B12 intervention on hematological parameters

| **Author, year** | **Country** | **Population** | **Intervention** | **Intervention duration** | ***n*** | **Hemoglobin pre-intervention (g/dL)** |  | **Hemoglobin post-intervention (g/dL)** | **Main findings relevant to vitamin B12 on hematological parameters** |
| --- | --- | --- | --- | --- | --- | --- | --- | --- | --- |
|  |  |  |  |  |  |  | ***n*** |  |  |
| Yajnik et al. 2007 [19] | India | Females (20-50y) | 100g cooked GLV + placebo (control) | Every alternate day for 6 weeks | 20 | 11.3 (10.4, 12.3) | 20 | 11.5 (10.3, 12.4) | *Hb*: significantly improved at 2 weeks but not 6 weeks  *MCV*: no significant change in vitamin B12 group |
|  |  |  | 100g cooked GLV + 500µg vitamin B12 (MC) |  | 20 | 11.9 (11.1, 12.5) | 20 | 11.9 (11.2, 13.1) |  |
| Chandelia et al. 2012 [20] | India | Children (0.5-5y) with anemia | 20mg Fe + FA (<2y, 2.6mg; >2y, 5.1mg) (control) | Daily for 2 months | 76 | 7.2 (0.7) | 70 | 9.9 (1.0) | *Hb*: significantly higher at 4 and 8 weeks in B12 group.  *MCV*: decreased in both groups but NS |
|  |  |  | (a) 20mg Fe + FA (<2y, 2.6mg; >2y, 5.1mg) + vitamin B12 (MC; <1y, 125μg; 1-2y, 250μg and >2y, 500μg)  (b) 20mg Fe + FA (<2y, 2.6mg; >2y, 5.1mg) + vitamin B12 injection (MC; <2y, 250μg and >2y, 500μg)  *Groups (a) and (b) combined for analysis* | Daily for 2 months | 66 | 6.9 (0.8) | 66 | 11.0 (0.8) |  |
|  |  |  |  | Days 1, 15 and 29 |  |  |  |  |  |
| Shahab-Ferdows et al. 2012 [21] | Mexico | Females (20-59y) | Placebo | Daily for 3 months | 62 | 14.0 (0.9) | 62 | -0.01 (-0.1 to 0.1) | *Hematological parameters*: no significant difference between groups |
|  |  |  | 1mg vitamin B12 IM followed by 500µg oral vitamin B12 (OH) |  | 70 | 13.8 (0.9) | 70 | -0.01 (-0.2 to 0.2) |  |
| Jenssen et al. 2013 [22] | Norway | Infants (6 weeks) | No treatment | Single dose  4 months | 53 | 12.9 (1.6) | 50 | 12.1 (1.0) | *Hematological parameters*: no significant difference between groups |
|  |  |  | 400μg vitamin B12 IM injection (OH) |  | 54 | 13.1 (2.1) | 52 | 12.0 (0.8) |  |
| Bansal et al. 2016 [23] | India | Adolescent girls (11-18y) with anemia | 100mg Fe + 500μg FA + placebo (control) | Weekly for 26 weeks | 184 | 10.7 (1.1) | 184 | 11.6 (1.1) | *Hb*: significantly improved in both groups.  *Anemia*: prevalence decreased in both groups by ~40% |
|  |  |  | 100mg Fe + 500μg FA + 500μg vitamin B12 for 6 weeks then 15μg for 20 weeks |  | 189 | 10.8 (0.9) | 189 | 11.7 (1.0) |  |
| Kumar et al. 2017 [12] | India | Children (6-30 months) | Placebo | Daily for 6 months | 64 | 10.1 (1.3) | 63 | 9.9 (1.4) | *Hb*: no significant difference between placebo and treatment groups |
|  |  |  | 1.8µg vitamin B12 |  | 65 | 9.8 (1.3) | 63 | 9.6 (1.3) |  |
|  |  |  | 150µg FA + 1.8µg vitamin B12  *One additional treatment arm of FA* |  | 66 | 10.2 (1.2) | 64 | 9.7 (1.6) |  |
| Gupta et al. 2021 [24] | India | Adolescent girls (12-19y) with anemia | 60mg Fe + 500μg FA + placebo (control) | Daily for 3 months | 246 | 10.5 (0.8) | 246 | 12.1 (1.0) | *Hb*: significantly higher response in control group.  *Anemia*: greater reduction in prevalence in control group (68% control *vs* 40% B12) |
|  |  |  | 60mg Fe + 500μg FA + 1000μg vitamin B12 (CN) |  | 514 | 10.3 (0.9) | 514 | 11.6 (0.9) |  |
| Yajnik et al. 2021 [25] | India | Children (10-13y) | Placebo bar | Daily for 4 months | 55 | 13.7 (13.4, 14.2) | 55 | 13.9 (13.2, 14.3) | *Hb*: no significant difference within any group |
|  |  |  | 2µg vitamin B12 fortified bar |  | 54 | 13.8 (13.1, 14.3) | 54 | 13.9 (13.4, 14.4) |  |
|  |  |  | 1.82µg vitamin B12 + MMN fortified bar |  | 55 | 13.7 (13.0, 14.1) | 55 | 13.5 (12.9, 14.2 |  |
|  |  | Adults (18-50y) | Placebo yogurt | Daily for 4 months | 40 | 11.5 (10.0, 12.4) | 40 | 11.9 (10.5, 13.0) | *Hb*: no significant difference within any group |
|  |  |  | 2µg vitamin B12 yogurt |  | 39 | 11.4 (10.4, 13.4) | 39 | 11.6 (10.2, 14.0) |  |
|  |  |  | 1 x 10^8^ cfu/g Propionibacterium yogurt |  | 39 | 11.2 (9.5, 12.0) | 39 | 11.3 (10.1, 12.3) |  |

Data presented as mean (SD), median (25^th^, 75^th^ percentile) or median change (range). CN, cyanocobalamin; FA, folic acid; Fe, iron; GLV, green leafy vegetables; Hb, hemoglobin; IFA, iron plus folic acid; IM, intramuscular; MC, methylcobalamin; MCV, mean cell volume; MMN, multiple micronutrients; NS, Not significant; OH, hydroxycobalamin

**Supplementary Table 5:** Summary of trials investigating the effects of zinc intervention on hematological parameters

| **Study** | **Country** | **Population** | **Intervention** | **Intervention duration** | ***n*** | **Hemoglobin pre-intervention (g/dL)** | ***n*** | **Hemoglobin post-intervention (g/dL)** | **Main findings relevant to zinc on hematological parameters** |
| --- | --- | --- | --- | --- | --- | --- | --- | --- | --- |
| Barffour et al. 2019  [26] | Laos | Children (6-23 months) | 7mg Zn | Daily for ~9 months | 738 | 10.8 (0.1) | 722 | 11.0 (0.0) | *Anemia*: No significant difference in anemia prevalence of Zn alone *vs* placebo group |
|  |  |  | 10mg Zn + 6mg Fe + 13 other micronutrients |  | 701 | 10.8 (0.1) | 689 | 11.1 (0.0) |  |
|  |  |  | 20mg/d Zn for 10 days per diarrheal episode |  | 764 | 10.8 (0.1) | 751 | 11.1 (0.0) |  |
|  |  |  | Placebo |  | 740 | 10.8 (0.1) | 726 | 11.1 (0.0) |  |
| Berger et al. 2006  [27] | Vietnam | Infants (4-7 months) | 10mg Fe | Daily for 6 months | 234 | 11.1 (11.7) | 195 | 13.2 (1.7) | *Hb*: No significant difference between Zn alone *vs* placebo group |
|  |  |  | 10mg Zn |  | 221 | 10.9 (1.4) | 190 | 11.8 (1.6) |  |
|  |  |  | 10mg Fe + 10mg Zn |  | 234 | 10.9 (1.4) | 184 | 12.9 (1.6) |  |
|  |  |  | Placebo |  | 226 | 10.8 (1.6) | 191 | 11.8 (1.6) |  |
| Dijkhizen et al. 2001  [28] | Indonesia | Infants (4 months) | 10mg Fe | Daily for 6 months | 120 | *N/A* | 90 | 11.5 (1.0) | *Anemia*: No significant difference in anemia prevalence of Zn alone (62%) *vs* placebo (66%) group |
|  |  |  | 10mg Zn |  | 119 |  | 97 | 10.6 (1.1) |  |
|  |  |  | 10mg Fe + 10 mg Zn |  | 120 |  | 74 | 11.0 (1.1) |  |
|  |  |  | Placebo |  | 119 |  | 87 | 10.6 (1.1) |  |
| Hanson et al. 2023 [29] | USA | Adults with chronic anemia | 50mg Zn sulfate | Daily for 12 months | 34 | 9.96 | 17 | 10.93 | *Hb*: Significantly improved in patients with Zn deficiency and chronic anemia |
| Kolsteren et al. 1999  [30] | Bangladesh | Women with anemia (15-45y) | 60mg Fe (FeSO4) | Daily for 60 days | 56 | 9.3 (1.0) | 56 | 10.6 (1.0) | *Hb*: Fe+vitamin A+Zn group had significantly increased Hb response than Fe alone group |
|  |  |  | 60mg Fe (FeSO4) +  200 000 IU vitamin A |  | 57 | 9.0 (1.2) | 57 | 10.5 (0.8) |  |
|  |  |  | 60mg Fe (FeSO_4_) +  200 000 vitamin A + 15mg Zn (zinc gluconate) |  | 58 | 8.9 (1.1) | 58 | 10.7 (1.0) |  |
| Nishiyama et al. 1998 [31] | Japan | Women with anemia (35-60y) | 100mg Fe citrate | Daily for 6 weeks | 15 | 10.8 (0.9) | 15 | 11.4 (0.7) | *Hb*: Fe alone and Zn alone groups had similar increase on Hb but not significantly. Fe+Zn group had statistically significant increase pre- and post-intervention |
|  |  |  | 35mg Zn gluconate |  | 21 | 11.0 (0.7) |  | 11.4 (0.7) |  |
|  |  |  | 100mg Fe citrate + 35mg Zn gluconate |  | 16 | 10.8 (1.1) |  | 12.8 (1.1) |  |
| Saaka et al. 2012 [32] | Ghana | Pregnant women (<16 weeks) | 40mg FeSO_4_ + 40mg Zn gluconate | Every other day for 18 weeks | 82 | Anemic:  9.7 (0.7) | 82 | Anemic:  10.9 (1.1) | *Hb*: Fe+Zn supplementation was more effective than Fe alone in raising Hb among iron deficient women |
|  |  |  |  |  | 113 | Non-anemic:  12.2 (0.9) | 113 | Non-anemic:  10.9 (1.3) |  |
|  |  |  | 40mg FeSO4 (control) |  | 72 | Anemic:  9.7 (0.7) | 72 | Anemic:  10.7 (1.1) |  |
|  |  |  |  |  | 122 | Non-anemic:  12.2 (1.1) | 122 | Non-anemic:  11.1 (1.1) |  |
| Silva et al. 2006 [33] | Brazil | Children (12-59 months receiving Fe fortified cow’s milk) | 10mg Zn sulfate | Daily for 4 months | 28 | 11.1 (0.9) | 28 | 11.8 (1.1) | *Anemia*: Zn group had statistically significant greater decrease of anemia prevalence (25%) than placebo group (6.6%) |
|  |  |  | Placebo |  | 30 | 11.6 (1.2) | 30 | 12.1 (0.7) |  |

Data presented as mean (SD). Fe, iron; Hb, hemoglobin; Zn, zinc.

**Supplementary Table 6:** Summary of review articles investigating the effects of zinc supplementation on anemia and/or hemoglobin concentrations

| **Author (Year)** | **Review type** | **Population(s)** | **Country** | **Studies (*n*)** | **Main findings** |
| --- | --- | --- | --- | --- | --- |
| Fischer et al. 2005 [34] | Narrative review | Pregnant women, infants, children | Nepal, Bangladesh, Indonesia, Mexico, Papua New Guinea, Chile, Belize, Peru | 10  Pregnant women (n=2)  Infants (n=3)  Children (n=5) | 9 out of 10 studies reported no effect of zinc supplementation on hemoglobin (no data shown) |
| Mayo-Wilson et al. 2014 [35] | Systematic review and meta-analysis | Children (6 months-12y) | 32 represented, most conducted in LMICs | 80 | No effect of zinc supplementation on hemoglobin (n=27 trials, n=6024 people, Fixed effects: SMD=-0.05 (-0.10 to 0.00), Heterogeneity I^2^, X^2^, p-value: 45%, 63.96 p=0.002) |
| Tam et al. 2020 [36] | Systematic review and meta-analysis | Children (<5y) | LMICs | 136 (n=31 on zinc supplementation) | Anemia risk was not reduced by zinc supplementation  (n=10 studies, RR 1.01 95% CI (0.96, 1.06), I^2^=0%, X^2^=7.23) |

**Supplementary Table 7:** Summary of trials investigating the effects of vitamin D intervention on hematological parameters

| **Study** | **Country** | **Population** | **Intervention** | **Duration** | ***n*** | **Hemoglobin pre-intervention (g/dL)** | ***n*** | **Hemoglobin post-intervention (g/dL)** | **Main findings** |
| --- | --- | --- | --- | --- | --- | --- | --- | --- | --- |
| O’Callaghan et al. 2023 [37] | Bangladesh | Pregnant women (17-24 weeks gestation) | Prenatal: postpartum doses:  0;0 IU | Weekly until 26 weeks postpartum | 203 | 10.6 (1.1) | 203 | N/A | *Ferritin*: Concentration at delivery similar between each intervention arm and placebo group in adjusted (n=998) and baseline ferritin-adjusted analyses (n=992, P>0.05) |
|  |  |  | 4200;0 IU cholecalciferol |  | 202 | 10.7 (1.2) | 202 |  |  |
|  |  |  | 16 800;0 IU cholecalciferol |  | 205 | 10.6 (1.1) | 205 |  |  |
|  |  |  | 28 000; 28 000 IU cholecalciferol |  | 388 | 10.7 (1.1) | 388 |  |  |
| Ahmad et al. 2019 [38] | UK | Premenopausal women (19-49y), Fe-deficient (plasma ferritin concentration <20 µg/L) | Fe-fortified (9mg) breakfast cereal + 1500 IU cholecalciferol | Daily for 8 weeks | 25 | 1.4 (1.2) | 20 | 1.4 (1.0 | *Hb, Hct*: Significantly improvement in Hb (P=0.037) and Hct concentration (P=0.032) significantly higher in vitamin D group *vs* placebo |
|  |  |  | Fe-fortified (9mg) breakfast cereal + placebo |  | 25 | 1.3 (1.5) | 24 | 1.3 (1.3) |  |
| Braithwaite et al. 2019 [39] | UK | Pregnant women (>18y, singleton pregnancy, 10-17 weeks gestation) | 1000 IU/d cholecalciferol | Daily from ~25 weeks until delivery) | 93 | N/A | 93 | N/A | *Ferritin*: No difference between groups by late pregnancy (P=0.94) |
|  |  |  | Placebo |  | 102 |  | 102 |  |  |
| Ernst et al. 2017 [40] | Germany | Men and women (18-79y) with congestive heart failure | 4000 IU cholecalciferol | Daily for 36 months | 85 | 14.2 (1.6) | 85 | 13.7 (1.8) | *Hb*: No significant difference in between groups (P=0.87) |
|  |  |  | Placebo |  | 87 | 14.2 (1.5) | 87 | 13.8 (1.9) |  |
| Ernst et al. 2016 [41] | Austria | Men and women with serum 25(OH)D concentration < 75 nmol/L and arterial hypertension | 2800 IU cholecalciferol | Daily for 8 weeks | 100 | 14.4 (1.3) | 93 | 14.3 (1.3) | *Hb*: No significant difference between groups (P=0.661) |
|  |  |  | Placebo |  | 100 | 14.4 (1.4) | 95 | 14.3 (1.3) |  |
| Kasprowicz et al. 2020 [42] | Poland | Adult male amateur runners | 10 000 IU cholecalciferol | Daily for 2 weeks | 10 | N/A | 8 | N/A | *Ferritin*: No significant difference between groups (pre-100 km run) (P>0.05) |
|  |  |  | Placebo |  | 10 |  | 9 |  |  |
| Madar et al. 2016 [43] | Norway | Male and females of South Asian, Middle East, or African descent (18-50y) | 400 IU cholecalciferol | Daily for 16 weeks | 85 | 13.7 (1.6) | 69 | 13.5 (1.6) | No significant difference in serum ferritin, Hb, iron, or transferrin saturation between groups (P>0.05) |
|  |  |  | 1000 IU cholecalciferol |  | 84 | 13.6 (1.6) | 74 | 13.5 (1.6) |  |
|  |  |  | Placebo |  | 82 | 13.6 (1.4) | 71 | 13.3 (1.4) |  |
| Smith et al. 2017 [44] | USA | Healthy adults | 250,000 IU cholecalciferol oral bolus | One-time oral bolus | 14 | N/A | 14 | N/A | *Ferritin*: No difference in ferritin concentrations between groups (P=0.44) |
|  |  |  | Placebo |  | 14 |  | 14 |  |  |
| Smith et al. 2018 [45] | USA | Mechanically ventilated critically ill adults | 250,000 IU total cholecalciferol enteral bolus | 5 days (5 equal doses over time period) | 9 | 10.3 (9.1, 11.7) | 9 | N/A | Hb: Significant difference in hemoglobin concentration in group supplemented with 500, 000 IU *vs* placebo, no significant difference in those supplemented with 250,000 IU *vs* placebo |
|  |  |  | 500,000 IU total cholecalciferol enteral bolus |  | 11 | 9.6 (8.5, 10.7) | 11 |  |  |
|  |  |  | Placebo |  | 10 | 8.7 (7.7, 9.8) | 10 |  |  |
| Sooragonda et al. 2015 [46] | India | Anemic patients (serum ferritin < 15 µg/L, ages 150-60 y) | 600,000 IU cholecalciferol IM | 12 weeks (one-time IM dose) | 15 | 9.7 (1.3) | 15 | 11.8 (1.5) | *Hb, ferritin*: No significant difference in Hb (P=0.6) or ferritin (P=0.2) between groups |
|  |  |  | Placebo |  | 15 | 9.2 (1.4) | 15 | 12.1 (1.5) |  |

Data presented as mean (SD) or geometric mean (95% CI). Hb, hemoglobin; Hct, hematocrit; IM, intramuscular; IU, international units; 25(OH)D, 25-hydroxyvitamin D.

**References:**

1.	Barnabé A, Aléssio ACM, Bittar LF, de Moraes Mazetto B, Bicudo AM, de Paula EV, et al. Folate, vitamin B12 and homocysteine status in the post-folic acid fortification era in different subgroups of the Brazilian population attended to at a public health care center. Nutrition Journal. 2015 Feb 19;14(1):19. doi:10.1186/s12937-015-0006-3

2.	Palchetti CZ, Steluti J, Sales CH, Fisberg RM, Marchioni DML. Folate and vitamin B12 status: temporal evaluation after mandatory fortification in Brazil. Eur J Clin Nutr. 2022 Sep;76(9):1266–72. doi:10.1038/s41430-022-01096-4 PubMed PMID: 35318452.

3.	Zhou Y, Wang A, Yeung LF, Qi YP, Pfeiffer CM, Crider KS. Folate and vitamin B12 usual intake and biomarker status by intake source in United States adults aged ≥19 y: NHANES 2007-2018. Am J Clin Nutr. 2023 Jul;118(1):241–54. doi:10.1016/j.ajcnut.2023.05.016 PubMed PMID: 37172826; PubMed Central PMCID: PMC10926004.

4.	Jones KS, Collins D, Meadows SR, Koulman A, Page P. National diet and nutrition survey data reveal a decline in folate status in the United Kingdom population between 2008 and 2019. Am J Clin Nutr. 2023 Dec;118(6):1182–91. doi:10.1016/j.ajcnut.2023.10.006 PubMed PMID: 37839706; PubMed Central PMCID: PMC10739772.

5.	Solvik BS, Strand TA, Kvestad I, Markhus MW, Ueland PM, McCann A, et al. Dietary intake and biomarkers of folate and Cobalamin status in Norwegian preschool children: the FINS-KIDS study. J Nutr. 2020 Jul 1;150(7):1852–8. doi:10.1093/jn/nxaa111 PubMed PMID: 32338756; PubMed Central PMCID: PMC7330454.

6.	Sundarakumar JS, Shahul Hameed SK, SANSCOG Study Team, Ravindranath V. Burden of vitamin D, vitamin B12 and folic acid deficiencies in an aging, rural Indian community. Front Public Health. 2021;9:707036. doi:10.3389/fpubh.2021.707036 PubMed PMID: 34540786; PubMed Central PMCID: PMC8446357.

7.	Mára M, Zivný J, Eretová V, Kvasnicka J, Kuzel D, Umlaufová A, et al. Changes in markers of anemia and iron metabolism and how they are influenced by antianemics in postpartum period. Acta Obstet Gynecol Scand. 2001 Feb;80(2):142–8. PubMed PMID: 11167209.

8.	Juarez-Vazquez J, Bonizzoni E, Scotti A. Iron plus folate is more effective than iron alone in the treatment of iron deficiency anaemia in pregnancy: a randomised, double blind clinical trial. BJOG. 2002 Sep;109(9):1009–14. doi:10.1111/j.1471-0528.2002.01378.x PubMed PMID: 12269674.

9.	Christian P, Shrestha J, LeClerq SC, Khatry SK, Jiang T, Wagner T, et al. Supplementation with micronutrients in addition to iron and folic acid does not further improve the hematologic status of pregnant women in rural Nepal. J Nutr. 2003 Nov;133(11):3492–8. doi:10.1093/jn/133.11.3492 PubMed PMID: 14608063.

10.	Hadler MCCM, Sigulem DM, Alves M de FC, Torres VM. Treatment and prevention of anemia with ferrous sulfate plus folic acid in children attending daycare centers in Goiânia, Goiás State, Brazil: a randomized controlled trial. Cad Saude Publica. 2008;24 Suppl 2:S259-271. doi:10.1590/s0102-311x2008001400011 PubMed PMID: 18670706.

11.	Rosado JL, González KE, Caamaño MDC, García OP, Preciado R, Odio M. Efficacy of different strategies to treat anemia in children: a randomized clinical trial. Nutr J. 2010 Sep 23;9:40. doi:10.1186/1475-2891-9-40 PubMed PMID: 20863398; PubMed Central PMCID: PMC2955680.

12.	Kumar T, Taneja S, Sachdev HPS, Refsum H, Yajnik CS, Bhandari N, et al. Supplementation of vitamin B12 or folic acid on hemoglobin concentration in children 6-36 months of age: A randomized placebo controlled trial. Clin Nutr. 2017 Aug;36(4):986–91. doi:10.1016/j.clnu.2016.07.002 PubMed PMID: 27486122.

13.	den Elzen WPJ, Westendorp RGJ, Frölich M, de Ruijter W, Assendelft WJJ, Gussekloo J. Vitamin B12 and folate and the risk of anemia in old age: the Leiden 85-Plus Study. Arch Intern Med. 2008 Nov 10;168(20):2238–44. doi:10.1001/archinte.168.20.2238 PubMed PMID: 19001201.

14.	Shobar RM, Velineni S, Keshavarzian A, Swanson G, DeMeo MT, Melson JE, et al. The Effects of Bowel Preparation on Microbiota-Related Metrics Differ in Health and in Inflammatory Bowel Disease and for the Mucosal and Luminal Microbiota Compartments. Clin Transl Gastroenterol. 2016 Feb;7(2):e143. doi:10.1038/ctg.2015.54 PubMed PMID: 26866392; PubMed Central PMCID: PMC4817412.

15.	Samuel TM, Duggan C, Thomas T, Bosch R, Rajendran R, Virtanen SM, et al. Vitamin B(12) intake and status in early pregnancy among urban South Indian women. Ann Nutr Metab. 2013;62(2):113–22. doi:10.1159/000345589 PubMed PMID: 23344013; PubMed Central PMCID: PMC4742783.

16.	Ramírez-Vélez R, Correa-Bautista JE, Martínez-Torres J, Meneses-Echávez JF, Lobelo F. Vitamin B12 concentrations in pregnant Colombian women: analysis of nationwide data 2010. BMC Pregnancy Childbirth. 2016 Feb 1;16:26. doi:10.1186/s12884-016-0820-4 PubMed PMID: 26832149; PubMed Central PMCID: PMC4736095.

17.	Chandyo RK, Kvestad I, Ulak M, Ranjitkar S, Hysing M, Shrestha M, et al. The effect of vitamin B12 supplementation during pregnancy on infant growth and development in Nepal: a community-based, double-blind, randomised, placebo-controlled trial. Lancet. 2023 May 6;401(10387):1508–17. doi:10.1016/S0140-6736(23)00346-X PubMed PMID: 37031691.

18.	Finkelstein JL, Fothergill A, Johnson CB, Guetterman HM, Bose B, Jabbar S, et al. Anemia and vitamin B-12 and folate status in women of reproductive age in Southern India: estimating population-based risk of neural tube defects. Curr Dev Nutr. 2021 May;5(5):nzab069. doi:10.1093/cdn/nzab069 PubMed PMID: 34027296; PubMed Central PMCID: PMC8128722.

19.	Yajnik CS, Lubree HG, Thuse NV, Ramdas LV, Deshpande SS, Deshpande VU, et al. Oral vitamin B12 supplementation reduces plasma total homocysteine concentration in women in India. Asia Pac J Clin Nutr. 2007;16(1):103–9. PubMed PMID: 17215186.

20.	Chandelia S, Chandra J, Narayan S, Aneja S, Chawla HM, Sharma S, et al. Addition of cobalamin to iron and folic acid improves hemoglobin rise in nutritional anemia. Indian J Pediatr. 2012 Dec;79(12):1592–6. doi:10.1007/s12098-012-0725-9 PubMed PMID: 22415494.

21.	Shahab-Ferdows S, Anaya-Loyola MA, Vergara-Castañeda H, Rosado JL, Keyes WR, Newman JW, et al. Vitamin B-12 supplementation of rural Mexican women changes biochemical vitamin B-12 status indicators but does not affect hematology or a bone turnover marker. J Nutr. 2012 Oct;142(10):1881–7. doi:10.3945/jn.112.165712 PubMed PMID: 22915298.

22.	Jenssen HB, Torsvik I, Ueland PM, Midttun Ø, Bjørke-Monsen AL. Biochemical signs of impaired cobalamin function do not affect hematological parameters in young infants: results from a double-blind randomized controlled trial. Pediatr Res. 2013 Sep;74(3):327–32. doi:10.1038/pr.2013.101 PubMed PMID: 23770919.

23.	Bansal PG, Toteja GS, Bhatia N, Vikram NK, Siddhu A. Impact of weekly iron folic acid supplementation with and without vitamin B12 on anaemic adolescent girls: a randomised clinical trial. Eur J Clin Nutr. 2016 Jun;70(6):730–7. doi:10.1038/ejcn.2015.215 PubMed PMID: 26695724.

24.	Gupta A, Kant S, Ramakrishnan L, Pandey RM, Khandelwal R, Kapil U, et al. Impact of daily-supervised administration of a package of iron and folic acid and vitamin B12 on hemoglobin levels among adolescent girls (12-19 years): a cluster randomized control trial. Eur J Clin Nutr. 2021 Nov;75(11):1588–97. doi:10.1038/s41430-021-00878-6 PubMed PMID: 33828241.

25.	Yajnik C, Kasture S, Kantikar V, Lubree H, Bhat D, Raut D, et al. Efficacy of B12 fortified nutrient bar and yogurt in improving plasma B12 concentrations-results from 2 double-blind randomized placebo controlled trials. Food Nutr Bull. 2021 Dec;42(4):480–9. doi:10.1177/03795721211025448 PubMed PMID: 34189981.

26.	Barffour MA, Hinnouho GM, Kounnavong S, Wessells KR, Ratsavong K, Bounheuang B, et al. Effects of daily zinc, daily multiple micronutrient powder, or therapeutic zinc supplementation for diarrhea prevention on physical growth, anemia, and micronutrient status in rural laotian children: a randomized controlled trial. J Pediatr. 2019 Apr;207:80-89.e2. doi:10.1016/j.jpeds.2018.11.022 PubMed PMID: 30580974; PubMed Central PMCID: PMC6448681.

27.	Berger J, Ninh NX, Khan NC, Nhien NV, Lien DK, Trung NQ, et al. Efficacy of combined iron and zinc supplementation on micronutrient status and growth in Vietnamese infants. Eur J Clin Nutr. 2006 Apr;60(4):443–54. doi:10.1038/sj.ejcn.1602336 PubMed PMID: 16306925.

28.	Dijkhuizen MA, Wieringa FT, West CE, Martuti S, Muhilal null. Effects of iron and zinc supplementation in Indonesian infants on micronutrient status and growth. J Nutr. 2001 Nov;131(11):2860–5. doi:10.1093/jn/131.11.2860 PubMed PMID: 11694609.

29.	Hanson ZD, Mirshahidi H, Brothers J, Mirshahidi S, Pham B, Samaeekia R, et al. Hemoglobin response to zinc supplementation in chronic kidney disease in patients with zinc deficiency and anemia. Blood. 2023 Nov 2;142(Supplement 1):3827. doi:10.1182/blood-2023-183066

30.	Kolsteren P, Rahman SR, Hilderbrand K, Diniz A. Treatment for iron deficiency anaemia with a combined supplementation of iron, vitamin A and zinc in women of Dinajpur, Bangladesh. Eur J Clin Nutr. 1999 Feb;53(2):102–6. doi:10.1038/sj.ejcn.1600684 PubMed PMID: 10099942.

31.	Nishiyama S, Irisa K, Matsubasa T, Higashi A, Matsuda I. Zinc status relates to hematological deficits in middle-aged women. J Am Coll Nutr. 1998 Jun;17(3):291–5. doi:10.1080/07315724.1998.10718762 PubMed PMID: 9627918.

32.	Saaka M. Combined iron and zinc supplementation improves haematologic status of pregnant women in upper west region of Ghana. Ghana Med J. 2012 Dec;46(4):225–33. PubMed PMID: 23661841; PubMed Central PMCID: PMC3645177.

33.	Silva APR, Vitolo MR, Zara LF, Castro CFS. Effects of zinc supplementation on 1- to 5-year old children. J Pediatr (Rio J). 2006;82(3):227–31. doi:10.2223/JPED.1480 PubMed PMID: 16738738.

34.	Fischer Walker C, Kordas K, Stoltzfus RJ, Black RE. Interactive effects of iron and zinc on biochemical and functional outcomes in supplementation trials. Am J Clin Nutr. 2005 Jul;82(1):5–12. doi:10.1093/ajcn.82.1.5 PubMed PMID: 16002793.

35.	Mayo-Wilson E, Imdad A, Junior J, Dean S, Bhutta ZA. Preventive zinc supplementation for children, and the effect of additional iron: a systematic review and meta-analysis. BMJ Open. 2014 Jun 19;4(6):e004647. doi:10.1136/bmjopen-2013-004647 PubMed PMID: 24948745; PubMed Central PMCID: PMC4067863.

36.	Tam E, Keats EC, Rind F, Das JK, Bhutta AZA. Micronutrient supplementation and fortification interventions on health and development outcomes among children under-five in low- and middle-income countries: a systematic review and meta-analysis. Nutrients. 2020 Jan 21;12(2):289. doi:10.3390/nu12020289 PubMed PMID: 31973225; PubMed Central PMCID: PMC7071447.

37.	O’Callaghan K, Qamar H, Gernand A, Onoyovwi A, Zlotkin S, Mahmud A, et al. Maternal prenatal, with or without postpartum, vitamin D3 supplementation does not improve maternal iron status at delivery or infant iron status at 6 months of age: secondary analysis of a randomised controlled trial. BMJ Nutr Prev Health. 2023;6:e000758.

38.	Ahmad Fuzi SF, Mushtaq S. Vitamin D3 supplementation for 8 weeks leads to improved haematological status following the consumption of an iron-fortified breakfast cereal: a double-blind randomised controlled trial in iron-deficient women. Br J Nutr. 2019 May;121(10):1146–57. doi:10.1017/S0007114519000412 PubMed PMID: 30819262.

39.	Braithwaite VS, Crozier SR, D’Angelo S, Prentice A, Cooper C, Harvey NC, et al. The effect of vitamin D supplementation on hepcidin, iron status, and inflammation in pregnant women in the United Kingdom. Nutrients. 2019 Jan 18;11(1):190. doi:10.3390/nu11010190 PubMed PMID: 30669280; PubMed Central PMCID: PMC6356300.

40.	Ernst JB, Prokop S, Fuchs U, Dreier J, Kuhn J, Knabbe C, et al. Randomized supplementation of 4000 IU vitamin D3 daily vs placebo on the prevalence of anemia in advanced heart failure: the EVITA trial. Nutr J. 2017 Aug 23;16(1):49. doi:10.1186/s12937-017-0270-5 PubMed PMID: 28835271; PubMed Central PMCID: PMC5569566.

41.	Ernst JB, Tomaschitz A, Grübler MR, Gaksch M, Kienreich K, Verheyen N, et al. Vitamin D supplementation and hemoglobin levels in hypertensive patients: a randomized controlled trial. Int J Endocrinol. 2016;2016:6836402. doi:10.1155/2016/6836402 PubMed PMID: 27006655; PubMed Central PMCID: PMC4781958.

42.	Kasprowicz K, Ratkowski W, Wołyniec W, Kaczmarczyk M, Witek K, Żmijewski P, et al. The effect of vitamin D3 supplementation on hepcidin, iron, and IL-6 responses after a 100 km ultra-marathon. Int J Environ Res Public Health. 2020 Apr 24;17(8):2962. doi:10.3390/ijerph17082962 PubMed PMID: 32344650; PubMed Central PMCID: PMC7215841.

43.	Madar AA, Stene LC, Meyer HE, Brekke M, Lagerløv P, Knutsen KV. Effect of vitamin D3 supplementation on iron status: a randomized, double-blind, placebo-controlled trial among ethnic minorities living in Norway. Nutr J. 2016 Aug 9;15(1):74. doi:10.1186/s12937-016-0192-7 PubMed PMID: 27506667; PubMed Central PMCID: PMC4977672.

44.	Smith EM, Alvarez JA, Kearns MD, Hao L, Sloan JH, Konrad RJ, et al. High-dose vitamin D3 reduces circulating hepcidin concentrations: A pilot, randomized, double-blind, placebo-controlled trial in healthy adults. Clin Nutr. 2017 Aug;36(4):980–5. doi:10.1016/j.clnu.2016.06.015 PubMed PMID: 27402475; PubMed Central PMCID: PMC5191989.

45.	Smith EM, Jones JL, Han JE, Alvarez JA, Sloan JH, Konrad RJ, et al. High-dose vitamin D3 administration is associated with increases in hemoglobin concentrations in mechanically ventilated critically ill adults: a pilot double-blind, randomized, placebo-controlled trial. JPEN J Parenter Enteral Nutr. 2018 Jan;42(1):87–94. doi:10.1177/0148607116678197 PubMed PMID: 29505145; PubMed Central PMCID: PMC5423855.

46.	Sooragonda B, Bhadada SK, Shah VN, Malhotra P, Ahluwalia J, Sachdeva N. Effect of vitamin D replacement on hemoglobin concentration in subjects with concurrent iron-deficiency anemia and vitamin D deficiency: a randomized, single-blinded, placebo-controlled trial. Acta Haematol. 2015;133(1):31–5. doi:10.1159/000357104 PubMed PMID: 25012612.
